# Incipient Sympatric Speciation and Evolution of Soil Bacteria Revealed by Metagenomic and Structured Non-Coding RNAs Analysis

**DOI:** 10.3390/biology11081110

**Published:** 2022-07-26

**Authors:** Sumit Mukherjee, Zhuoran Kuang, Samrat Ghosh, Rajesh Detroja, Gon Carmi, Sucheta Tripathy, Danny Barash, Milana Frenkel-Morgenstern, Eviatar Nevo, Kexin Li

**Affiliations:** 1 State Key Laboratory of Grassland Agro-Ecosystem, College of Ecology, Lanzhou University, Lanzhou 730050, China; kuangzhr21@lzu.edu.cn; 2 Department of Computer Science, Ben-Gurion University, Beer-Sheva 8410501, Israel; dbarash@bgu.ac.il; 3 Cancer Genomics and BioComputing of Complex Diseases Lab, Azrieli Faculty of Medicine, Bar-Ilan University, Safed 1311502, Israel; rajesh007detroja@gmail.com (R.D.); gon.carmi@biu.ac.il (G.C.); milana.morgenstern@biu.ac.il (M.F.-M.); 4 Institute of Evolution, University of Haifa, Mount Carmel, Haifa 3498838, Israel; nevo@research.haifa.ac.il; 5 Computational Genomics Laboratory, Department of Structural Biology and Bioinformatics, CSIR-Indian Institute of Chemical Biology, Kolkata 700054, India; samrat.ghosh2010@gmail.com (S.G.); tsucheta@iicb.res.in (S.T.); 6 Academy of Scientific and Innovative Research (AcSIR), Ghaziabad 201009, India

**Keywords:** sympatric speciation, evolution plateau, metagenomics, genetic divergence, structured non-coding RNA, riboswitches

## Abstract

**Simple Summary:**

The microevolutionary dynamics of soil bacteria under microclimatic differences are largely unexplored in contrast to our improving knowledge of their vast diversity. In this study, we performed a comparative metagenomic analysis of two sharply divergent rocks and soil types at the Evolution Plateau (EP) in eastern Upper Galilee, Israel. We have identified the significant differences in bacterial taxonomic diversity, functions, and patterns of RNA-based gene regulation between the bacteria from two different soil types. Furthermore, we have identified several species with a significant genetic divergence of the same species between the two soil types, highlighting the soil bacteria’s incipient sympatric speciation.

**Abstract:**

Soil bacteria respond rapidly to changes in new environmental conditions. For adaptation to the new environment, they could mutate their genome, which impacts the alternation of the functional and regulatory landscape. Sometimes, these genetic and ecological changes may drive the bacterial evolution and sympatric speciation. Although sympatric speciation has been controversial since Darwin suggested it in 1859, there are several strong theoretical or empirical evidences to support it. Sympatric speciation associated with soil bacteria remains largely unexplored. Here, we provide potential evidence of sympatric speciation of soil bacteria by comparison of metagenomics from two sharply contrasting abutting divergence rock and soil types (Senonian chalk and its rendzina soil, and abutting Pleistocene basalt rock and basalt soil). We identified several bacterial species with significant genetic differences in the same species between the two soil types and ecologies. We show that the bacterial community composition has significantly diverged between the two soils; correspondingly, their functions were differentiated in order to adapt to the local ecological stresses. The ecologies, such as water availability and pH value, shaped the adaptation and speciation of soil bacteria revealed by the clear-cut genetic divergence. Furthermore, by a novel analysis scheme of riboswitches, we highlight significant differences in structured non-coding RNAs between the soil bacteria from two divergence soil types, which could be an important driver for functional adaptation. Our study provides new insight into the evolutionary divergence and incipient sympatric speciation of soil bacteria under microclimatic ecological differences.

## 1. Introduction

Diverse microbiome populations play a critical role in the environments ranging from soil to human and animal guts [1,2]. The soil microbiome plays a functional role in carbon cycling, decomposition of organic matter, nutrient transformation, maintenance of ecosystem sustainability [3], and supporting plant growth while providing vital ecosystems [2,4]. Vice versa, the microbiome could be affected by their depending environments [5]. Correspondingly, different strains of the same species can be significantly distinct in their gene content and single-nucleotide polymorphisms (SNPs) [6,7]. These strain-level differences have shed light on understanding adaptation, evolution, and sympatric speciation of the microbiomes. For example, differentiated ecologies drove the population subdivision of ocean bacteria [8] and also the presence of subpopulations in ancient marine bacteria [9]. Emerging shreds of evidence support that changes in ecology and environmental patterns drive the evolution of microbial species [10,11,12,13]. A recent study demonstrated the rapid strain level evolution of soil bacteria in response to climate changes [14]. To quickly adapt to the new environment, bacteria rapidly mutate their gene sequences [15,16] and non-coding RNAs [17], which can generate new functionality and regulatory mechanisms. This mechanism could promote the continuous emergence of new strains [18,19]. The continuous genetic changes and fitness stress associated with the new ecology may generate new bacterial species.

However, the origin of species has been controversial since first suggested by Darwin [20]. The allopatric speciation common model hypothesized that populations evolve into new species when geographically isolated, unaffected by the homogenizing process of gene flow, and thus, they can accumulate sufficient genetic divergence and reproductive isolation to evolve into new species. By contrast, the sympatric speciation (SS) model hypothesized that a new species might originate as a local variety [20], and in general, one species splits into two sister species without any physical barriers to block gene flow between the two populations. In modern terminology, a new species can evolve within a smaller space characterized by free breeding meta-populations with gene flow, provided there are divergent ecologies in the speciation theater [21]. The SS model was and is controversial since suggested and still considered rare by most biologists, though mounting evidence in eukaryotes, both theoretical and empirical [22,23,24,25], does indicate that new species indeed can originate despite ongoing gene flow (Table S1). The SS model becomes even more enigmatic in bacteria whose systematics is still problematic. We proposed a new paradigm shift to incorporate ecology and divergent genetics in bacterial systematics [26] based on our two microsite evolution models, the microclimatic microsite model [27,28,29,30], and the geological-edaphic microsite model [24,25,31]. In the first model designated “Evolution Canyon” (EC), a tropical, hot and dry, savannoid biome abuts with temperate, cool and humid, forested biome across a few hundred meters. In the second model, two different rocks and soil types abut at “Evolution Plateau” (EP) in eastern Upper Galilee: Senonian chalk abuts with Pleistocene volcanic basalt. Both models, the microclimatic and edaphic, demonstrated hotspots of SS, primarily unfolded in eukaryotes from bacteria to mammals, with only one case of prokaryotes, in the soil bacterium *Bacillus simplex* [32]. Here, we asked whether SS is relevant in prokaryotes, as we and others (Table S1) highlighted in eukaryotes. Our criterion of identifying sympatric speciation is based on our new ecological paradigm linked with little to medium genetic change between the progenitor and derivative species speciating sympatrically in the new ecology. Shotgun metagenomic sequencing and biocomputing are widely applied to study the genetic diversity of the uncultured microbial communities in diverse environments [1]. Recently, strain-level analysis of metagenomes has shown that shotgun metagenomic sequencing has the potential to understand strain-level heterogeneity within bacterial genomes and between microbiome communities in contrast to only the sequencing of 16S ribosomal RNA [33]. All these current approaches to detect strain-level variations are dependent on the availability of a collection of microbial reference genomes to identify SNPs and quantify gene content in microbial species [33,34].

**Figure 1:**
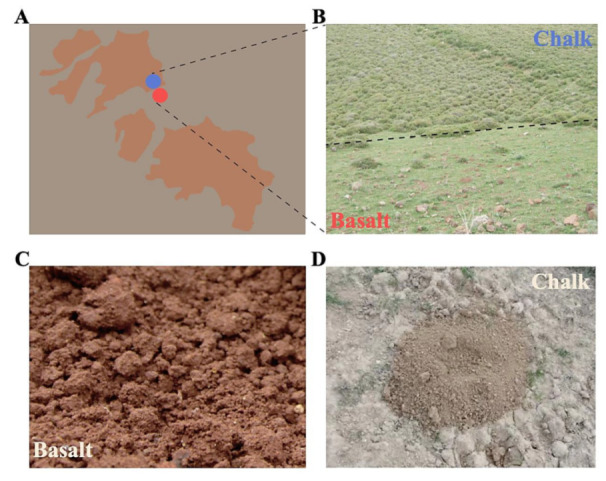
Geological map and ecological differences. (**A**) Geological map including the senonian chalk soil and the abutting derivative Plio-Pleistocence basalt soil, which is like reddish basaltic islands in pale chalk ocean. (**B**) The contrasting plants with only 28% of the same plant species in the different abutting soils. (**C**) The clayey wetter and milder basalt soil, and (**D**) the drier and stressful chalk soil, with a mound of the mole rat *Spalax galili* chalk.

To study the bacterial evolution, including the SS within the microbiomes of different soils, we compared the metagenomes from two sharply divergent rocks and soil types (chalk weathering into rendzina soil and volcanic basalt into basalt soil) at the EP, in the eastern Upper Galilee Israel (Figure 1A). The basalt is clayey, wetter, and muddy with low permeability (Figure 1B); in contrast, the chalk is drier and barren with better ventilation (Figure 1C). Ca^2+^ is the most abundant element in the chalk soil (Figure 1D), followed by Si^4+^, Al^3+^, Fe^3+^, and Mg^2+^, and Si^4+^ is the most abundant chemical in the abutting contrasting basalt soil, followed by Al^3+^, Fe^3+^, and Ca^2+^. Al^3+^, Fe^3+^, and Ca^2+^ contents are significantly different between the two soils [35]. Thus, basalt is generally acidic, while chalk is slightly alkaline [36]. Such contrasting ecologies without any physical barriers lead to population divergences with gene flow, including bacteria [32], wild barley [37], and blind mole rat [24,25,31,38,39,40,41]. This present study demonstrated that the bacterial community composition has significantly diverged between the chalk and basalt soil types. Furthermore, we observed that the bacteria from two different soil types have significant differences in functions and preferences of structured non-coding RNAs, highlighting the evolution of functional diversity to adapt locally to new ecological stresses. Moreover, remarkably, we identified the incipient sympatric speciation in soil bacteria as we identified in eukaryotes across life in our evolution microsites, based on divergent ecologies, with ongoing gene flow, suggesting that natural selection can overrule the homogenizing effect of gene flow in both eukaryotes and prokaryotes [42].

## 2. Materials and Methods

### 2.1. Sampling, DNA Isolation, and Sequencing

A total of 12 soil samples were collected for chalk (6 samples) and abutting derivative basalt (6 samples) from “Evolution Plateau” (EP) in the eastern Upper Galilee Israel in January 2016 and emerged into liquid nitrogen immediately in the field. The samples were from one line and one sample every 50 meter. All the samples were transported to a company for DNA isolation and sequencing. DNA was isolated from each soil sample by using QIAmp DNA stool mini kit (Qiagen, Hilden, Germany). Qualified DNAs were sonicated to about 350 bp fragments; after purification, end-repair, A-tailing, and adaptor ligation were carried out and followed by PCR amplification. After quality control, libraries of each sample were sequenced with 150 bp paired-end reads. A total of ~50 million 150 bp paired-end raw reads were generated from the shotgun metagenomic sequencing of each sample.

### 2.2. Quality Control, Reads Assembly, Binning, Refinement, Assessment, and Annotation

Quality control (QC) of raw sequenced reads was performed using FastQC v0.11.9 [43], and, according to the QC report, hugely imbalanced 10 bases from start and N’s from start/end of the reads were trimmed off using cutadapt [44] tool. Simultaneously, reads were assembled using metaSPAdes v3.15.4 [45]. After assembly, assembled contigs were binned using Metabat2 v2.15 [46], and the quality of each bin was further improved using Binning_refiner. Binning followed by refinement of bins able to be recovered metagenome-assembled genomic reads (MAGs) (based on universal standards, i.e., contamination < 10% and completeness ≥ 50% (medium), and contamination < 5% and completeness ≥ 90% (high)). Finally, each bin quality was evaluated using CheckM v1.2.0 [47], and according to standard criteria, bins were selected. The taxonomy of each selected bin was assigned using GTDB-tk tool v2.1.0 [48], and annotations of these bins were carried out using Prokka v1.14.5 [49].

### 2.3. Taxonomic Compositions of Basalt and Chalk Sample

Kraken2 [50] was used for taxonomic profiling, and the relative abundance was estimated by Bracken [51]. Next, taxonomic and functional data tables were downloaded and fed into the STAMP v2.1.3 (Statistical analysis of metagenomic profile) software [52] for statistical validation. The species with significant differences were identified by LEfSe (linear discriminant analysis effect size, LDA score > 2) [53] on the website (http://huttenhower.sph.harvard.edu/galaxy/, v1.0, accessed on 19 May 2022).

### 2.4. Function Compositions of Basalt and Chalk Sample

High-quality unassembled reads were first uploaded onto the MG-RAST server [54] for functional profiling, and the subsystem database was selected with default parameters. In order to obtain a more accurate contig for the construction of non-redundant gene catalogs, we use MEGAHIT [55] to assemble the reads; next, we use Prodigal [56] to predict coding sequences (CDS, >100 bp) of assembled contig and use CD-HIT [57] (-c 0.95 -d 0 -aL 0.9 -uL 0.05 -aS 0.9) to obtain a non-redundant gene catalog. KoafmKOALA [58] was performed to annotate functional genes against the Kofam database. KEGG enrichment was accomplished by ReporterScore [59]. Carbohydrate active enzymes (CAZyomes) were identified by dbCAN2 [60]. The relative gene abundance was calculated as follows:

Step 1: Calculation of the copy number of each gene: b_i_ = x_i_/L_i_;
Step 2: Calculation of the relative abundance of gene i: a_i_ = b_i_/∑b_i_:
  a_i_: the relative abundance of gene I;
  b_i_: the copy number of gene i from sample N;
  L_i_: the length of gene i;
  x_i_: the number of mapped reads.

The calculations mentioned above were performed with our custom Python scripts.

### 2.5. Analysis of Structured Noncoding RNAs in the Metagenome-Assembled Genomic Reads (MAGs) Sequences

Riboswitches are the most common form of structured noncoding RNAs in bacteria [61,62,63,64,65]. The Rfam 12.0 [66] database contains sequence and structural information of more than 40 classes of riboswitches. We have downloaded all the covariance models of each riboswitch class from Rfam. Then, the cmsearch program of the Infernal 1.1 package [67] was used to identify the riboswitches from the metagenome-assembled genomic reads (MAGs). Furthermore, the detected riboswitches were again confirmed with the Riboswitch Scanner [68]. To obtain a local minimum energy structure, aligned consensus structure generated using the covariance model was passed to RNAfold from the Vienna RNA package 2.0 [69] as enforced structure constraints. This enables generating the potential riboswitch structure where the local minimum energy and covariance model are both considered [63,70]. FORNA from Vienna RNA package was used to visualize the riboswitch structure.

### 2.6. Metagenomic Single Nucleotide Variants (SNVs) Analysis

In order to calculate sympatric speciation in bacteria based on single nucleotide variant (SNV), first, human contaminations were removed by mapping trimmed reads to the human reference genome (hg38) using BWA tool [71]. Next, unmapped (non-human) reads were mapped to the CDS sequences of RefSoil+ database [2], which contains 888 bacteria and 34 archaea. Furthermore, mapping of RefSoil+ was subjected to the metaSNV pipeline to calculate pairwise distance between chalk and basalt samples based on population SNVs [34].

### 2.7. Single Nucleotide Polymorphisms (SNPs) Analysis

For the most abundant species, their genomes were downloaded from NCBI as references. Reads after removing human contaminations were mapped against these genomes by BWA, and we use GATK4 [72] to call hard-filtered SNPs with default parameters. Principal component analysis (PCA) was performed by PLINK [73]. Neighbor-joining (NJ) tree was accomplished by TreeBeST [74]. *F_ST_* and nucleotide diversity (π) were calculated by VCFtools [75]. Furthermore, a genetic distance matrix was generated by PLINK, then visualization of genetic network was finished by R packages [76] (“netview”, “network”, “igraph”, “sna”, “visNetwork”, “threejs”, and “networkD3”).

## 3. Results

### 3.1. Metagenome Assembly

A total of ~16 GB of raw reads from the shotgun metagenomic sequencing has been generated (Table S2), and after trimming the low-quality bases, about 15 Gb of clean reads were retained for each of the six samples from basalt and six samples from chalk soils. After assembly, an average of 850,000 contigs (>300 bp) was generated for each sample. Statistical information of assembled contigs (Table S3) shows that these assemblies were sufficient for the taxonomical and functional analysis of our study. We performed a Spearman correlation analysis to understand whether the differences between the two groups of samples (basalt and chalk) are more significant than the biological repeats (samples from the same group). We found that the differences between biological repeats are significantly smaller than between the samples from another group (Figure S1), which signify the significant differences between the samples from basalt and chalk.

### 3.2. Bacterial Community Composition

**Figure 2:**
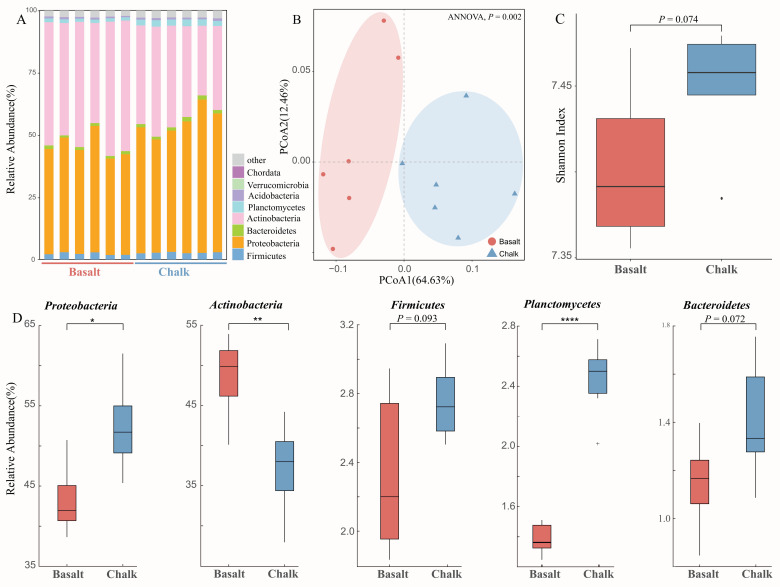
The taxonomy analysis of soil microbiomes and relative abundance of the two soil types. (**A**) Bacteria composition, which shows the most abundant species are from Actinobacteria and Proteobacteria. (**B**) Principal component analysis shows the bacterial composition of the two soil types was clearly separated. (**C**) Comparison of community diversity measured by Shannon diversity, showing it was higher in the arid chalk than that in the clayey wetter and milder basalt soils. (**D**) The relative abundance of the five dominant phyla. In this figure, *, **, and **** indicates the significance levels *p* < 0.05, *p* < 0.01, and *p* < 0.0001 accordingly.

Bacteria were the most dominant kingdom in both of the soils, followed by Archaea and Eukaryota. For the bacterial community, 58 phyla, 98 classes, 212 orders, 462 families, 1791 genera, and 6413 species were detected by metagenomic sequencing from 12 soil samples. We identified 32 phyla, 56 classes, 135 orders, 289 families, 981 genera, and 3103 species that were significantly different between basalt and chalk (Welch’s *t*-test, *p* < 0.05). Among the bacterial phyla, Proteobacteria and Actinobacteria were found to be the most abundant phyla both in chalk soil as well as in basalt soil, accounting for over 90% of the total population of all the phyla, followed by Bacteroidetes, Acidobacteria, Verrucomicrobia, and Chordata (Figure 2A). Bacterial composition was separated into two clusters in PCoA (Figure 2B), and it is significantly higher in the more arid chalk soil (Figure 2C). Actinobacteria was significantly higher in basalt than in chalk (Figure 2D); in contrast, Proteobacteria, Firmicutes, Planctomycetes, and Bacteroidetes were higher in chalk than in basalt. High abundances of Proteobacteria and Actinobacteria indicate that these phyla played important roles in the soil bacterial communities providing basic functions related to the biogeochemical cycle. Moreover, we performed a *t*-test (paired) and found significant differences in a relative phylum abundance of Proteobacteria (*p* = 0.011), Actinobacteria (*p* = 4.6 × 10^−3^), Planctomycetes (*p* = 3.16 × 10^−5^), Chordata (*p* = 1.93 × 10^−4^), and Cyanobacteria (*p* = 9.87 × 10^−4^) (Figure 2A). Further genus-level analysis indicates that Streptomyces, Micromonospora, Bradyrhizobium, Kribbella, Actinoplanes, and Amycolatopsis were enriched in basalt, while in chalk, Sphingobium, Burkholderia, Xanthomonas, Mesorhizobium, and Rubrobacter were enriched (Figure S2). Species with the most differences in abundance between basalt and chalk, revealed by linear discriminant analysis effect size (LEfSe) (Table S4), are: *Xanthomonas euvesicatoria*, *Sphingomonas sp.* MM-1 (enriched in chalk) and *Bradyrhizobium icense*, *Kribbella flavida* (enriched in basalt).

### 3.3. Functional Analysis of Metagenomic Data

**Figure 3:**
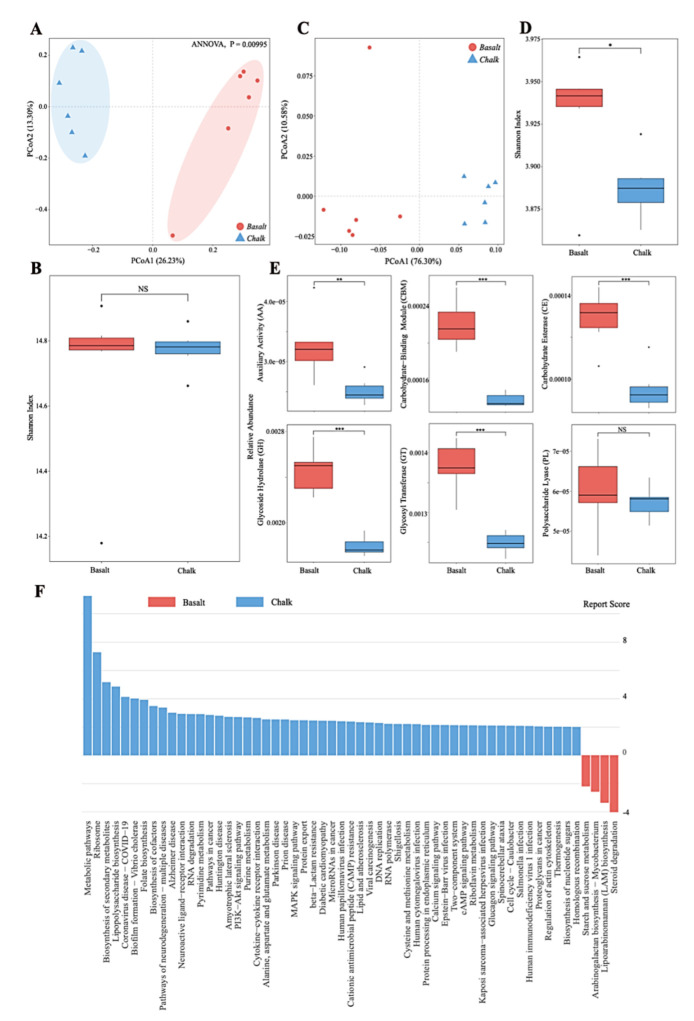
Functional composition of the two soil type metagenomics. (**A**) Principal component analysis shows the function composition of the two soil types was clearly separated. (**B**) There was no significant difference based on the Shannon index of non-redundant gene sets. (**C**) Principal component analysis shows that the comparison of carbohydrate-active enzymes (CAZyomes) composition of the two soil types was clearly separated. (**D**) The diversity of CAZYomes in basalt is higher. (**E**) Comparison of abundances of different CAZYomes families between basalt and chalk. (**F**) KEGG pathways with significant differences between basalt and chalk. In this figure, *, **, and *** indicates the significance levels *p* < 0.05, *p* < 0.01, and *p* < 0.001; and NS indicates not significant accordingly.

Principal coordinate analysis (PCoA) based on a non-redundant gene catalog revealed the functional compositions of basalt and chalk are significantly different (Figure 3A, ANNOVA, *p* = 0.0095). There was no significant difference in the Shannon index of the two functional genes (Figure 3B). CAZyomes (carbohydrate-active enzymes) compositions of basalt and chalk are different (Figure 3C, ANNOVA, *p* = 0.0040), with a higher Shannon index in basalt. Specifically (Figure 3D), basalt contains more Auxiliary Activity (AA), Carbohydrate−Binding Module (CBM), Carbohydrate Esterase (CE), Glycoside Hydrolase (GH), and GlycosylTransferase (GT). GH13, GH92, GH5, GH18, and CBM32 were enriched in basalt, while in chalk, GT51, GH13, GT84, GT35, and GT9 were enriched (Figure S3). The significantly higher KEGG pathways (Figure 3E) in basalt included: “Metabolic pathways”, “Ribosome”, “Biosynthesis of secondary metabolites”, “Lipopolysaccharide biosynthesis”, “Folate biosynthesis”, “Purine metabolism”, and some immune- and disease-related pathways, such as “Alzheimer disease”, “Parkinson disease”, and “Prion disease”. The significantly higher KEGG pathways (Figure 3F) in chalk included: “Steroid degradation”, “Lipoarabinomannan (LAM) biosynthesis”, “Arabinogalactan biosynthesis—Mycobacterium”, and “Starch and sucrose metabolism”.

The taxonomic assignments of the chalk and basalt soil samples were obtained by analyzing assembled contigs and the gene prediction and the classification of the potential functional genes via Clusters of Orthologous (COG) analysis. We summarized the results into four categories: information storage and processing (cluster I); cellular processes and signaling (cluster II); metabolism (cluster III); and poorly characterized function (cluster IV). Mostly, cluster III was dominant in both chalk and basalt soil samples which is related to the growth of microbial communities. Cluster II was the next most dominant in all the samples, followed by clusters IV and I (Figure S4). Functional annotation at level 1 revealed that genes related to “carbohydrates”, “co-factor, vitamins, prosthetic groups, pigments”, “RNA metabolism”, “Virulence, Disease and Defense”, and “Metabolism of aromatic compounds” were highly dominant in both chalk and basalt microbial communities (Figure S5). Functional annotation at level 2 demonstrated that genes related to “Di- and oligosaccharides”, “Biotin”, and “Phage and prophages” are most enriched in basalt, while genes related to “Resistance to antibiotics and toxic compounds” and “transcription” are most enriched in basalt (Figure S6). Therefore, the functional analysis demonstrated that the enriched processes are important for the functional adaptation of the microbes to survive in the stress associated with the specific soil environments.

### 3.4. Analysis of Structured Noncoding RNAs in Metagenome-Assembled Genomic Reads (MAGs) Sequences

**Figure 4:**
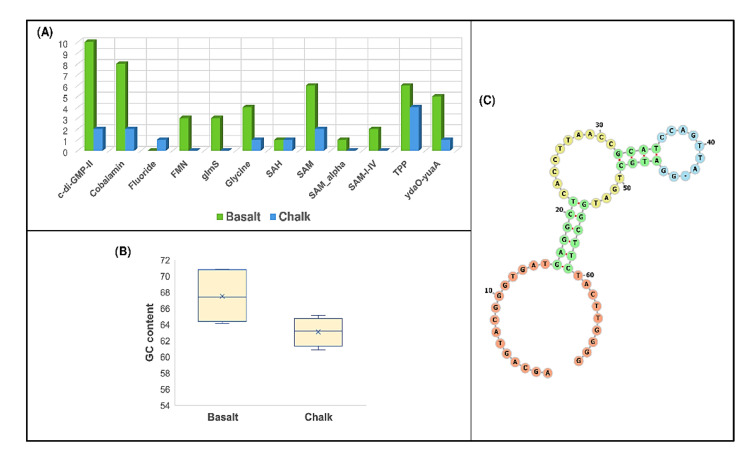
(**A**) Riboswitch distributions in the metagenomic assembled genomes (MAGs) from basalt and chalk samples. (**B**) The differences in GC content in the identified TPP riboswitches between basalt and chalk samples. (**C**) Secondary structure of fluoride sensing riboswitch identified in the MAGs from the chalk sample.

Bacterial structured noncoding RNAs play vital roles in several biological processes such as gene regulation, signaling, RNA processing, and protein synthesis [77]. Among these, the most common groups of structured noncoding RNAs in bacteria are riboswitches [61]. Riboswitches are the cis-regulatory structural RNA sensors found in the 5’ UTR of bacterial genes and control the expression of the associated genes/operons [78]. There are more than 40 different classes of riboswitch classes discovered so far, which are based on the type of ligand bind with the aptamer domain of specific riboswitches [61,78,79]. Understanding the abundance of specific riboswitch classes in bacteria can provide much deeper functional insights by identifying the metabolic pathway in which it participates and the conditions in which it is expressed. Therefore, we performed the riboswitch analysis to understand the abundance of riboswitch classes in the MAGs from basalt and chalk samples. We identified more riboswitches in the basalts than in chalk (Figure 4A), indicating that the preference for RNA-based gene regulation could be important for their evolutionary adaptation in the soil of the basalt region. Riboswitches are highly evolutionary conserved at both their sequence and structural levels. Specific mutations in riboswitches could affect the riboswitch conformation by causing a global rearrangement [17,80,81]. In [17], a mutational study was performed at an “Evolution Canyon”, and in [80], the rational design was exemplified by mutations predicted by energy minimization [69,81,82] and conformational switching was presented by using energy dot plots that can be analyzed in a variety of ways (e.g., as in [83]). Affecting riboswitches by mutations shows that sequence level variations of a particular riboswitch class that can significantly explain the differences between basalt and chalk bacterial groups could be useful for understanding the beneficial fitness effect in the specific group. For this purpose, we analyzed the TPP riboswitches, where six hits were found in basalt and four hits in chalk. We observed that TPP riboswitches detected in the MAGs from basalt have significantly higher GC content than MAGs in chalk (Avg_GC Basalt = 67.50, AVG_GC Chalk = 63.09, *p*-value = 0.0287) (Figure 4B). This observation indicates that GC content variation could shape the regulatory divergence of bacteria to survive in the face of different ecological stresses associated with the chalk and basalt region at Evolution Plateau (EP). Although we found that riboswitches are more abundant in the basalt sample than in chalk, we have detected Fluoride riboswitch in the MAGs of the chalk sample, which is absent in the basalt sample. Figure 4C represents the fluoride riboswitch structure detected in the MAGs from the chalk sample. The fluoride riboswitch is important for the bacterial defense mechanism in counteracting against the high fluoride toxicity by regulating downstream genes that encode putative fluoride transporters, enzymes that are known to be inhibited by fluoride [84]. Therefore, the presence of fluoride riboswitch in the bacterial MAGs of the chalk sample indicates that chalk soil could contain higher fluoride concentration, and some bacterial species use fluoride riboswitches to regulate the expression of proteins that alleviate the deleterious effects of fluoride. In conclusion, the diversity within the riboswitch-based gene regulation in between the bacteria of chalk and basalt samples indicates that structured noncoding RNAs are the potential driver for generating bacterial adaptation in new environments, which could play a significant role in bacterial evolution, including the sympatric speciation.

### 3.5. Genetic Divergence between the Two Microsites

**Figure 5:**
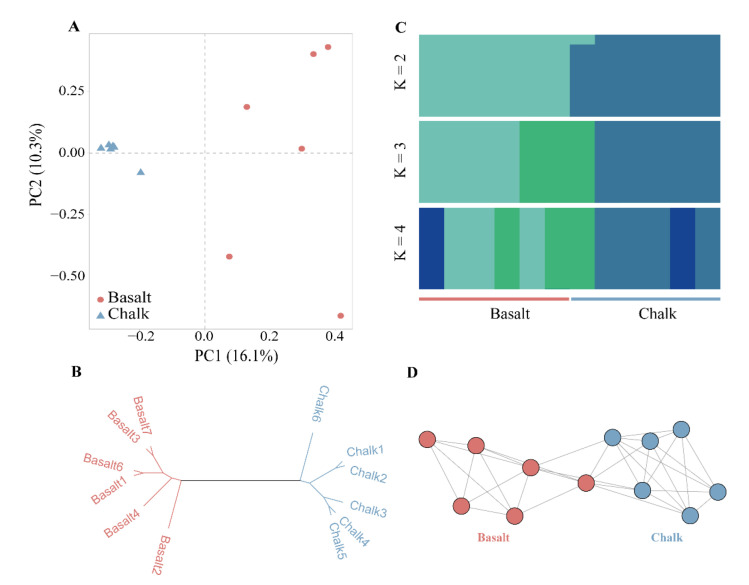
Sympatric divergence of the single bacteria of *Streptomyces lividans*. (**A**) Principal component analysis shows samples from basalt clustered together, and samples from chalk were in one cluster. (**B**) Phylogenetic tree of the chalk and abutting basalt populations. (**C**) Population structure analysis. (**D**) Genetic network analysis.

We have observed a significant difference in functional enrichment and noncoding structured RNAs between the bacteria from chalk and basalt samples. We hypothesized that the bacterial community evolved in two soil types to adapt to local ecological stress, where some bacterial species might be involved in SS. Sympatric speciation could explain the origination of new species from the ancestral species, while both are found in different ecological abutting contrasts. Although, in most cases, the complete genome information could not be mapped from metagenomic data, we can map and compare the large segment of the genome sequence of particular species to check if they are significantly different between two samples, which could help to infer the potential sympatric speciation in bacteria. To look at the potential sympatric speciation between the chalk and basalt soil samples, we performed the mapping to the reference database of soil bacteria and archaea containing the CDS sequences of 888 bacteria and 34 archaea. Interestingly, we found that most of the sympatric species were also found to be highly abundant at the genus level. Species-level pairwise distance based on single nucleotide variants (SNVs) between the chalk and basalt populations was calculated using the metaSNV pipeline [34], and it showed a significant difference in the same species between the two soil types (Figure S7). In PCoA, based on pairwise distance, we have identified more than 20 species showing clear-cut separate clusters (Figure S7). Furthermore, these species also have differences in SNP composition between chalk and basalt (Figure 5 and Figures S8–S25), PCA (Figure 5A and Figures S8A–S25A), phylogenetic tree (Figure 5B and Figures S8B–S25B), population STRUCTURE analysis (Figure 5C), and genetic networking (Figure 5D) showed separate genetic clusters of basalt and chalk, suggesting sympatric divergence. The genetic distance between the chalk and basalt soil populations of *Streptomyces lividans* measured by *F*_ST_ is 0.058, suggesting differentiation with gene flow. The nucleotide diversity (π) for basalt and chalk is 3.6 × 10^−3^ and 2.8 × 10^−3^, respectively. *F*_ST_ and nucleotide diversity (π) of other species were also calculated for these species based on SNPs (Table S5). However, this analysis failed to classify if they are new strains or new species, as retrieving complete genome sequence information of all these species was not possible from the metagenomic data. However, this finding indicates that several bacterial species change their genomic sequences to evolve and adapt to a new environment that could drive the generation of new species. Further experimental studies on sequencing each of the genomes of these bacteria and their phylogenomic analysis could identify many new bacterial species, which might be generated from the SS.

## 4. Discussion

SS has been controversial since Darwin suggested it (Darwin 1859). The key problem is whether they have allopatric history, and rare cases of SS admitted were from islands or small places [85]. In the current study, the SS arena, dubbed EP, was formed by a volcanic eruption around one Mya in chalk regions, which is like a basaltic island floating on a chalk ocean (Figure 1). The isolated basaltic island precludes the allopatric possibility, especially in the abutting regions, which is like the case of palm from abutting contrasting soils [86,87]. Our previous studies identified numerous SS events in EC and EP. Several SS across life, from bacteria to mammals, have been identified at EC, including the soil bacterium *Bacillus simplex* [32], *Hordeum spontaneum* [37], wild emmer wheat [88], *Triticum dicoccoides* [89], crucifer *Ricotia Lunaria* [90], *Oryzaphilus surinamensis* [91], etc. Similarly, several SS cases were identified from the EP [24,25,39,40], which was suggested to be an SS hotspot [92].

The central questions of bacterial ecology and evolution require a method to consistently demarcate, from the vast and diverse set of bacterial cells within a natural community, the groups playing ecologically distinct roles (ecotypes). Because of a lack of theory-based guidelines, current methods in bacterial systematics fail to divide the bacterial domain of life into meaningful units of ecology and evolution [26,93]. We introduce a sequence-based approach (“ecotype simulation”) to model the evolutionary dynamics of bacterial populations and to identify ecotypes within a natural community, focusing here on two Bacillus clades surveyed from the EC in Israel. This approach has identified multiple ecotypes within traditional species, with each predicted to be an ecologically distinct lineage; many such ecotypes were confirmed to be ecologically distinct, with specialization to different canyon slopes with different solar exposures.

In this study, high-throughput shotgun metagenomics sequencing and advanced bioinformatics algorithms enabled us to investigate microbial abundance and diversity under two sharply contrasting abutting divergent soil types. For example, microbial abundance at the phylum level shows that Actinobacteria was significantly highly abundant in basalt soil, while Bacteroidetes, Gemmatimonadetes, Firmicutes, and Verrucomicrobia were significantly highly abundant in chalk soil (Figure 2). The Actinobacteria are significantly higher in basalt soil, and Proteobacteria are significantly higher in chalk soil (Figure 2D), which is similar to a previous study [94] and was explained by the lower water availability in chalk than in basalt [94]. The basalt soil in Israel is typically slightly acidic [36]. Microbial abundance at the genus level shows that Streptomyces, Micromonospora, Kribbella, Microbacterium, and Frankia were significantly abundant in basalt soil samples. Firmicutes are higher in the dry chalk (Figure 2D), which is probably because the mild water stress could increase the relative abundance [95], and lower abundance members of the Firmicutes could facilitate bioremediation of acid basalt soil [96], where Gemmatimonas were significantly highly abundant in chalk soil samples.

Furthermore, we observed significant differences in functional enrichment between the bacteria from chalk and basalt soil. We found that most of the COG annotations were involved in microbial metabolism (Figure S4), and the relative abundance of cluster III (metabolism) was the highest among the four clusters. These functional differences help the bacterial community in specific soil adapt to the local ecology. Next, to check if there were any differences in non-coding RNA-based gene regulations, we performed the riboswitch analysis in the bacteria from two different soil types. Interestingly, we observed the differences in preferences of different metabolite sensing riboswitch classes. Furthermore, we detected differences in the GC content of highly conserved TPP riboswitch sequences between the bacteria from chalk and basalt soils, which indicates that these differences were generated due to the adaptive mutations that could have a significant role in the bacterial response to specific ecologies. All these findings decipher that the differential patterns of non-coding structural RNAs could alter the regulatory patterns of the bacteria, which could drive the soil bacterial evolution in microclimatic ecological differences.

Furthermore, we have also identified several sympatric species between the chalk and basalt soil populations, and many of them were also found to be highly abundant at the genus level in both populations. Overall, this study opens a new paradigm of soil bacterial evolution and sympatric speciation. Furthermore, our finding suggests that mutations in the functional genes and in the structured non-coding RNA are continuous processes that could help the soil bacterial community evolve continuously and adapt to ecological changes. The divergence of each species between the chalk and basalt soils showed clearly separate clusters, which were caused by the contrasting ecological stresses, either water availability or pH values, or both. Although there is no physical barrier between the two soils, the strong selection would overrule the gene flow between them, and the cumulated mutations facilitated speciation between them, just as in other species from the same speciation arena [24,25], which were proved to be SS. Since Evolution Canyons (ECs) and Evolution Plateaus (Eps) are numerous on our planet, SS appears to be a common model of the origin of species. Clearly, microsites divergent ecologically, geologically, edaphically, climatically, abiotically, and biotically are numerous across the planet; hence, SS, first hypothesized by Darwin, is proved to be a common model of speciation, not only in Israel but globally.

## 5. Conclusions

Soil bacteria tend to evolve rapidly in response to new environmental stress. However, the evolution of soil bacteria under microclimatic ecological differences is largely unexplored. In this study, we performed a comparative metagenomic analysis of two sharply divergent soil types and investigated the evolution of soil bacteria. Our findings elucidate the significant divergence of bacterial taxonomic compositions between two soil types. Furthermore, we found that the bacterial community from two soil types significantly differ in functional enrichment and preferences in RNA-based gene regulations. Hence, these findings suggested that the bacterial community diverged and functionally evolved to adapt to local ecological stress. Next, we detected several soil bacterial species have significant genetic divergence between the two soil types, which could support their evolution towards sympatric speciation. Thus, our study provided detailed insights into the soil bacterial evolution and incipient sympatric speciation under microclimatic ecological differences and encouraged further research in this area to uncover the evolutionary patterns of soil bacteria in the face of new ecological stresses.

## Data Availability

Not applicable.

## Supplementary Materials

The following supporting information can be downloaded at: https://www.mdpi.com/article/10.3390/biology11081110/s1, Figure S1: Spearman correlations between all the samples from basalt and chalk; Figure S2: Level of significance for relative abundances of all Genera in soil samples; Figure S3: Level of significance for relative abundances of all CaZy in soil samples; Figure S4: Functional categories for COGs; Figure S5: Functional group comparison of basalt and chalk microbial communities by SEED subsystems at level 1 using STAMP. STAMP indicates Statistical Analysis of Metagenomic Profiles; Figure S6: Functional group comparison of basalt and chalk microbial communities by SEED subsystems at level 2 using STAMP. STAMP indicates Statistical Analysis of Metagenomic Profiles; Figure S7: PCA projection of pairwise distance between chalk and basalt samples based on population SNVs; Figures S8–S25: Sympatric divergence of the individual bacteria. (A) Principal component analysis shows samples from basalt clustered together, and samples from chalk were in one cluster. (B) Phylogenetic tree of the chalk and abutting basalt populations. Table S1: List of articles on sympatric species; Table S2: Statistics of raw sequenced reads; Table S3: Statistics of assembled contigs; Table S4: Species identified with LEfSe that differed significantly in basalt and chalk, LDA SCORE (log10) > 2; Table S5: Sympatric divergence of the single bacteria of most 19 abundant bacteria, *F_ST_*, and nucleotide diversity (π) were calculated.

## References

[1] Sharpton T.J. (2014). An introduction to the analysis of shotgun metagenomic data. Front. Plant Sci..

[2] Dunivin T.K., Choi J., Howe A., Shade A. (2019). RefSoil+: A Reference Database for Genes and Traits of Soil Plasmids. mSystems.

[3] Altieri M.A. (1999). The ecological role of biodiversity in agroecosystems. Agric. Ecosyst. Environ..

[4] Choi J., Yang F., Stepanauskas R., Cardenas E., Garoutte A., Williams R., Flater J., Tiedje J.M., Hofmockel K.S., Gelder B. (2017). Strategies to improve reference databases for soil microbiomes. ISME J..

[5] Allison S.D., Martiny J.B.H. (2008). Resistance, resilience, and redundancy in microbial communities. Proc. Natl. Acad. Sci. USA.

[6] Greenblum S., Carr R., Borenstein E. (2015). Extensive strain-level copy-number variation across human gut microbiome species. Cell.

[7] Zhu A., Sunagawa S., Mende D.R., Bork P. (2015). Inter-individual differences in the gene content of human gut bacterial species. Genome Biol..

[8] Shapiro B.J., Friedman J., Cordero O.X., Preheim S.P., Timberlake S.C., Szabó G., Polz M.F., Alm E.J. (2012). Population genomics of early events in the ecological differentiation of bacteria. Science.

[9] Kashtan N., Roggensack S.E., Rodrigue S., Thompson J.W., Biller S.J., Coe A., Ding H., Marttinen P., Malmstrom R.R., Stocker R. (2014). Single-cell genomics reveals hundreds of coexisting subpopulations in wild Prochlorococcus. Science.

[10] Nguyen J., Lara-Gutiérrez J., Stocker R. (2021). Environmental fluctuations and their effects on microbial communities, populations and individuals. FEMS Microbiol. Rev..

[11] Vicuña R., González B. (2021). The microbial world in a changing environment. Rev. Chil. Hist. Nat..

[12] Collins S., Boyd P.W., Doblin M.A. (2020). Evolution, Microbes, and Changing Ocean Conditions. Ann. Rev. Mar. Sci..

[13] Scanlan P.D. (2019). Microbial evolution and ecological opportunity in the gut environment. Proc. R. Soc. B Boil. Sci..

[14] Chase A.B., Weihe C., Martiny J.B.H. (2021). Adaptive differentiation and rapid evolution of a soil bacterium along a climate gradient. Proc. Natl. Acad. Sci. USA.

[15] Massey R.C., Rainey P.B., Sheehan B.J., Keane O.M., Dorman C.J. (1999). Environmentally constrained mutation and adaptive evolution in Salmonella. Curr. Biol..

[16] Foster P.L., Lee H., Popodi E., Townes J.P., Tang H. (2015). Determinants of spontaneous mutation in the bacterium Escherichia coli as revealed by whole-genome sequencing. Proc. Natl. Acad. Sci. USA.

[17] Barash D., Sikorski J., Perry E.B., Nevo E., Nudler E. (2006). Adaptive mutations in RNA-based regulatory mechanisms: Computational and experimental investigations. Isr. J. Ecol. Evol..

[18] Graf M., Haas T., Müller F., Buchmann A., Harm-Bekbenbetova J., Freund A., Nieß A., Persicke M., Kalinowski J., Blombach B. (2019). Continuous Adaptive Evolution of a Fast-Growing Corynebacterium glutamicum Strain Independent of Protocatechuate. Front. Microbiol..

[19] Espejo R.T., García K., Plaza N. (2017). Insight into the origin and evolution of the Vibrio parahaemolyticus pandemic strain. Front. Microbiol..

[20] Darwin C. (1859). On the Origin of Species by Means of Natural Selection, or the Preservation of Favoured Races in the Struggle for Life.

[21] Coyne J.A. (2011). Speciation in a small space. Proc. Natl. Acad. Sci. USA.

[22] Wang Y., Qiao Z., Mao L., Li F., Liang X., An X., Zhang S., Liu X., Kuang Z., Wan N. (2022). Sympatric speciation of the spiny mouse from Evolution Canyon in Israel substantiated genomically and methylomically. Proc. Natl. Acad. Sci. USA.

[23] Li K., Wang H., Cai Z., Wang L., Xu Q., Lövy M., Wang Z., Nevo E. (2016). Sympatric speciation of spiny mice, Acomys, unfolded transcriptomically at Evolution Canyon, Israel. Proc. Natl. Acad. Sci. USA.

[24] Li K., Wang L., Knisbacher B.A., Xu Q., Levanon E.Y., Wang H., Frenkel-Morgenstern M., Tagore S., Fang X., Bazak L. (2016). Transcriptome, genetic editing, and microRNA divergence substantiate sympatric speciation of blind mole rat, Spalax. Proc. Natl. Acad. Sci. USA.

[25] Li K., Hong W., Jiao H., Wang G.D., Rodriguez K.A., Buffenstein R., Zhao Y., Nevo E., Zhao H. (2015). Sympatric speciation revealed by genome-wide divergence in the blind mole rat Spalax. Proc. Natl. Acad. Sci. USA.

[26] Koeppel A., Perry E.B., Sikorski J., Krizanc D., Warner A., Ward D.M., Rooney A.P., Brambilla E., Connor N., Ratcliff R.M. (2008). Identifying the fundamental units of bacterial diversity: A paradigm shift to incorporate ecology into bacterial systematics. Proc. Natl. Acad. Sci. USA.

[27] Nevo E. (1995). Asian, African and European biota meet at “Evolution Canyon” Israel: Local tests of global biodiversity and genetic diversity patterns. Proc. R. Soc. B Biol. Sci..

[28] Nevo E. (2012). “Evolution Canyon,” a potential microscale monitor of global warming across life. Proc. Natl. Acad. Sci. USA.

[29] Nevo E. (2009). Evolution in action across life at “evolution canyons”, Israel. Trends Evol. Biol..

[30] Nevo E. (2014). Evolution of wild emmer wheat and crop improvement. J. Syst. Evol..

[31] Hadid Y., Tzur S., Pavlíček T., Šumbera R., Šklíba J., Lövy M., Fragman-Sapir O., Beiles A., Arieli R., Raz S. (2013). Possible incipient sympatric ecological speciation in blind mole rats (Spalax). Proc. Natl. Acad. Sci. USA.

[32] Sikorski J., Nevo E. (2005). Adaptation and incipient sympatric speciation of Bacillus simplex under microclimatic contrast at “Evolution Canyons” I and II, Israel. Proc. Natl. Acad. Sci. USA.

[33] Nayfach S., Rodriguez-Mueller B., Garud N., Pollard K.S. (2016). An integrated metagenomics pipeline for strain profiling reveals novel patterns of bacterial transmission and biogeography. Genome Res..

[34] Costea P.I., Munch R., Coelho L.P., Paoli L., Sunagawa S., Bork P. (2017). metaSNV: A tool for metagenomic strain level analysis. PLoS ONE.

[35] Bian J., Cui L., Wang X., Yang G., Huo F., Ling H., Chen L., She K., Du X., Levi B. (2020). Genomic and Phenotypic Divergence in Wild Barley Driven by Microgeographic Adaptation. Adv. Sci..

[36] Polyakov A., Beharav A., Avivi A., Nevo E. (2004). Mammalian microevolution in action: Adaptive edaphic genomic divergence in blind subterranean mole-rats. Proc. R. Soc. B Biol. Sci..

[37] Li K., Ren X., Song X., Li X., Zhou Y., Harlev E., Sun D., Nevo E. (2020). Incipient sympatric speciation in wild barley caused by geological-edaphic divergence. Life Sci. Alliance.

[38] Li K., Zhang S., Song X., Weyrich A., Wang Y., Liu X., Wan N., Liu J., Lovy M., Cui H. (2020). Genome evolution of blind subterranean mole rats: Adaptive peripatric versus sympatric speciation. Proc. Natl. Acad. Sci. USA.

[39] Lövy M., Šklíba J., Hrouzková E., Dvoráková V., Nevo E., Šumbera R. (2015). Habitat and burrow system characteristics of the blind mole rat spalax galili in an area of supposed sympatric speciation. PLoS ONE.

[40] Lövy M., Šklíba J., Šumbera R., Nevo E. (2017). Soil preference in blind mole rats in an area of supposed sympatric speciation: Do they choose the fertile or the familiar?. J. Zool..

[41] Lövy M., Šumbera R., Heth G., Nevo E. (2020). Presumed ecological speciation in blind mole rats: Does soil type influence mate preferences?. Ethol. Ecol. Evol..

[42] Nevo E. (2011). Evolution under environmental stress at macro- and microscales. Genome Biol. Evol..

[43] Andrews S. (2010). FastQC: A quality control tool for high throughput sequence data. Babraham Bioinforma.

[44] Martin M. (2011). Cutadapt removes adapter sequences from high-throughput sequencing reads. EMBnet. J..

[45] Nurk S., Meleshko D., Korobeynikov A., Pevzner P.A. (2017). MetaSPAdes: A new versatile metagenomic assembler. Genome Res..

[46] Kang D.D., Froula J., Egan R., Wang Z. (2015). MetaBAT, an efficient tool for accurately reconstructing single genomes from complex microbial communities. PeerJ.

[47] Parks D.H., Imelfort M., Skennerton C.T., Hugenholtz P., Tyson G.W. (2015). CheckM: Assessing the quality of microbial genomes recovered from isolates, single cells, and metagenomes. Genome Res..

[48] Chaumeil P.A., Mussig A.J., Hugenholtz P., Parks D.H. (2020). GTDB-Tk: A toolkit to classify genomes with the genome taxonomy database. Bioinformatics.

[49] Seemann T. (2014). Prokka: Rapid prokaryotic genome annotation. Bioinformatics.

[50] Wood D.E., Lu J., Langmead B. (2019). Improved metagenomic analysis with Kraken. Genome Biol..

[51] Lu J., Breitwieser F.P., Thielen P., Salzberg S.L. (2017). Bracken: Estimating species abundance in metagenomics data. PeerJ Comput. Sci..

[52] Parks D.H., Tyson G.W., Hugenholtz P., Beiko R.G. (2014). STAMP: Statistical analysis of taxonomic and functional profiles. Bioinformatics.

[53] Segata N., Izard J., Waldron L., Gevers D., Miropolsky L., Garrett W.S., Huttenhower C. (2011). Metagenomic biomarker discovery and explanation. Genome Biol..

[54] Meyer F., Paarmann D., D’Souza M., Olson R., Glass E.M., Kubal M., Paczian T., Rodriguez A., Stevens R., Wilke A. (2008). The metagenomics RAST server—A public resource for the automatic phylogenetic and functional analysis of metagenomes. BMC Bioinform..

[55] Li D., Liu C.M., Luo R., Sadakane K., Lam T.W. (2015). MEGAHIT: An ultra-fast single-node solution for large and complex metagenomics assembly via succinct de Bruijn graph. Bioinformatics.

[56] Hyatt D., Chen G.L., LoCascio P.F., Land M.L., Larimer F.W., Hauser L.J. (2010). Prodigal: Prokaryotic gene recognition and translation initiation site identification. BMC Bioinform..

[57] Fu L., Niu B., Zhu Z., Wu S., Li W. (2012). CD-HIT: Accelerated for clustering the next-generation sequencing data. Bioinformatics.

[58] Aramaki T., Blanc-Mathieu R., Endo H., Ohkubo K., Kanehisa M., Goto S., Ogata H. (2020). KofamKOALA: KEGG Ortholog assignment based on profile HMM and adaptive score threshold. Bioinformatics.

[59] Bäckhed F., Roswall J., Peng Y., Feng Q., Jia H., Kovatcheva-Datchary P., Li Y., Xia Y., Xie H., Zhong H. (2015). Dynamics and stabilization of the human gut microbiome during the first year of life. Cell Host Microbe.

[60] Zhang H., Yohe T., Huang L., Entwistle S., Wu P., Yang Z., Busk P.K., Xu Y., Yin Y. (2018). DbCAN2: A meta server for automated carbohydrate-active enzyme annotation. Nucleic Acids Res..

[61] McCown P.J., Corbino K.A., Stav S., Sherlock M.E., Breaker R.R. (2017). Riboswitch diversity and distribution. RNA.

[62] Barrick J.E., Breaker R.R. (2007). The distributions, mechanisms, and structures of metabolite-binding riboswitches. Genome Biol..

[63] Mukherjee S., Das Mandal S., Gupta N., Drory-Retwitzer M., Barash D., Sengupta S. (2019). RiboD: A comprehensive database for prokaryotic riboswitches. Bioinformatics.

[64] Nudler E., Mironov A.S. (2004). The riboswitch control of bacterial metabolism. Trends Biochem. Sci..

[65] Mukherjee S., Barash D., Sengupta S. (2017). Comparative genomics and phylogenomic analyses of lysine riboswitch distributions in bacteria. PLoS ONE.

[66] Nawrocki E.P., Burge S.W., Bateman A., Daub J., Eberhardt R.Y., Eddy S.R., Floden E.W., Gardner P.P., Jones T.A., Tate J. (2015). Rfam 12.0: Updates to the RNA families database. Nucleic Acids Res..

[67] Nawrocki E.P., Eddy S.R. (2013). Infernal 1.1: 100-fold faster RNA homology searches. Bioinformatics.

[68] Mukherjee S., Sengupta S. (2016). Riboswitch Scanner: An efficient pHMM-based web-server to detect riboswitches in genomic sequences. Bioinformatics.

[69] Lorenz R., Bernhart S.H., Höner Zu Siederdissen C., Tafer H., Flamm C., Stadler P.F., Hofacker I.L. (2011). ViennaRNA Package 2. Algorithms Mol. Biol..

[70] Mukherjee S., Retwitzer M.D., Barash D., Sengupta S. (2018). Phylogenomic and comparative analysis of the distribution and regulatory patterns of TPP riboswitches in fungi. Sci. Rep..

[71] Li H., Durbin R. (2009). Fast and accurate short read alignment with Burrows-Wheeler transform. Bioinformatics.

[72] McKenna A., Hanna M., Banks E., Sivachenko A., Cibulskis K., Kernytsky A., Garimella K., Altshuler D., Gabriel S., Daly M. (2010). The genome analysis toolkit: A MapReduce framework for analyzing next-generation DNA sequencing data. Genome Res..

[73] Yang J., Lee S.H., Goddard M.E., Visscher P.M. (2011). GCTA: A tool for genome-wide complex trait analysis. Am. J. Hum. Genet..

[74] Ruan J., Li H., Chen Z., Coghlan A., Coin L.J.M., Guo Y., Hériché J.K., Hu Y., Kristiansen K., Li R. (2008). TreeFam: 2008 Update. Nucleic Acids Res..

[75] Danecek P., Auton A., Abecasis G., Albers C.A., Banks E., DePristo M.A., Handsaker R.E., Lunter G., Marth G.T., Sherry S.T. (2011). The variant call format and VCFtools. Bioinformatics.

[76] Steinig E.J., Neuditschko M., Khatkar M.S., Raadsma H.W., Zenger K.R. (2016). Netview p: A network visualization tool to unravel complex population structure using genome-wide SNPs. Mol. Ecol. Resour..

[77] Bastet L., Dub A., Mass E., Lafontaine D.A. (2011). New insights into riboswitch regulation mechanisms. Mol. Microbiol..

[78] Breaker R.R. (2011). Prospects for riboswitch discovery and analysis. Mol. Cell.

[79] Serganov A., Nudler E. (2013). A Decade of Riboswitches. Cell.

[80] Barash D., Gabdank I. (2010). Energy minimization methods applied to riboswitches: A perspective and challenges. RNA Biol..

[81] Barash D., Churkin A. (2011). Mutational analysis in RNAs: Comparing programs for RNA deleterious mutation prediction. Brief. Bioinform..

[82] Markham N.R., Zuker M. (2008). UNAFold: Software for nucleic acid folding and hybridization. Methods Mol. Biol..

[83] Ivry T., Michal S., Avihoo A., Sapiro G., Barash D. (2009). An image processing approach to computing distances between RNA secondary structures dot plots. Algorithms Mol. Biol..

[84] Baker J.L., Sudarsan N., Weinberg Z., Roth A., Stockbridge R.B., Breaker R.R. (2012). Widespread genetic switches and toxicity resistance proteins for fluoride. Science.

[85] Savolainen V., Anstett M.C., Lexer C., Hutton I., Clarkson J.J., Norup M.V., Powell M.P., Springate D., Salamin N., Baker W.J. (2006). Sympatric speciation in palms on an oceanic island. Nature.

[86] Papadopulos A.S.T., Igea J., Dunning L.T., Osborne O.G., Quan X., Pellicer J., Turnbull C., Hutton I., Baker W.J., Butlin R.K. (2019). Ecological speciation in sympatric palms: Genetic map reveals genomic islands underlying species divergence in Howea. Evolution.

[87] Papadopulos A.S.T., Igea J., Smith T.P., Hutton I., Baker W.J., Butlin R.K., Savolainen V. (2019). Ecological speciation in sympatric palms: Demographic analyses support speciation of Howea in the face of high gene flow. Evolution.

[88] Nevo E., Korol A., Beiles A., Fahima T. (2002). Evolution of Wild Emmer and Wheat Improvement: Population Genetics, Genetic Resources, and Genome Organization of Wheat’s Progenitor, Triticum Dicoccoides.

[89] Wang H., Yin H., Jiao C., Fang X., Wang G., Li G., Ni F., Li P., Su P., Ge W. (2020). Sympatric speciation of wild emmer wheat driven by ecology and chromosomal rearrangements. Proc. Natl. Acad. Sci. USA.

[90] Qian C., Yan X., Yin H., Fan X., Yin X., Sun P., Li Z., Nevo E., Ma X.F. (2018). Transcriptomes Divergence of Ricotia lunaria Between the Two Micro-Climatic Divergent Slopes at “Evolution Canyon” I, Israel. Front. Genet..

[91] Hong W., Li K., Sharaf K., Song X., Pavlìcek T., Zhao H., Nevo E. (2021). Genome-wide analysis revisits incipient sympatric and allopatric speciation in a beetle. Isr. J. Ecol. Evol..

[92] Nevo E. (2021). Evolution Canyons model: Biodiversity, adaptation, and incipient sympatric ecological speciation across life: A revisit. New Horizons in Evolution.

[93] Perry E.B., Krizanc D., Rooney A.P., Sikorski J., Nevo E., Cohan F.M. (2006). Identifying the fundamental units of diversity among Bacillus isolates from “Evolution Canyons” III. Isr. J. Ecol. Evol..

[94] Takebayashi S., Narihiro T., Fujii Y., Hiraishi A. (2007). Water Availability is a Critical Determinant of a Population Shift from Proteobacteria to Actinobacteria during Start-Up Operation of Mesophilic Fed-Batch Composting. Microbes Environ..

[95] Zhang R., Chen L., Niu Z., Song S., Zhao Y. (2019). Water stress affects the frequency of Firmicutes, Clostridiales and Lysobacter in rhizosphere soils of greenhouse grape. Agric. Water Manag..

[96] Gupta A., Dutta A., Sarkar J., Panigrahi M.K., Sar P. (2018). Low-abundance members of the firmicutes facilitate bioremediation of soil impacted by highly acidic mine drainage from the Malanjkhand copper project, India. Front. Microbiol..

