# Supplementary material for: Incipient Sympatric Speciation and Evolution of Soil Bacteria Revealed by Metagenomic and Structured Non-Coding RNAs Analysis
Source: Biology (Basel). 2022 Jul 26;11(8):1110. doi: 10.3390/biology11081110 (PMC9331176; doi:10.3390/biology11081110)
Supplement: Supplementary file 1 [file biology-11-01110-s001.zip › Supplementary Figure S6.pdf]

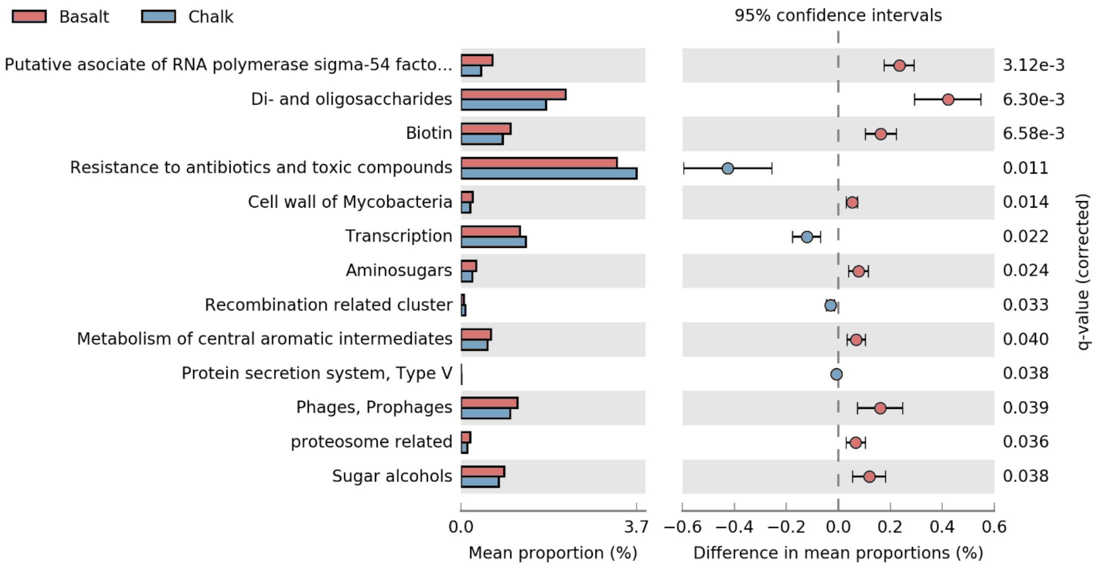

Figure S5. Functional group comparison of Basalt and Chalk microbial communities by SEED subsystems at level 2 using STAMP. STAMP indicates Statistical Analysis of Metagenomic Profiles.
