# Supplementary figures and images for: Incipient Sympatric Speciation and Evolution of Soil Bacteria Revealed by Metagenomic and Structured Non-Coding RNAs Analysis
Source: Biology (Basel). 2022 Jul 26;11(8):1110. doi: 10.3390/biology11081110 (PMC9331176; doi:10.3390/biology11081110)

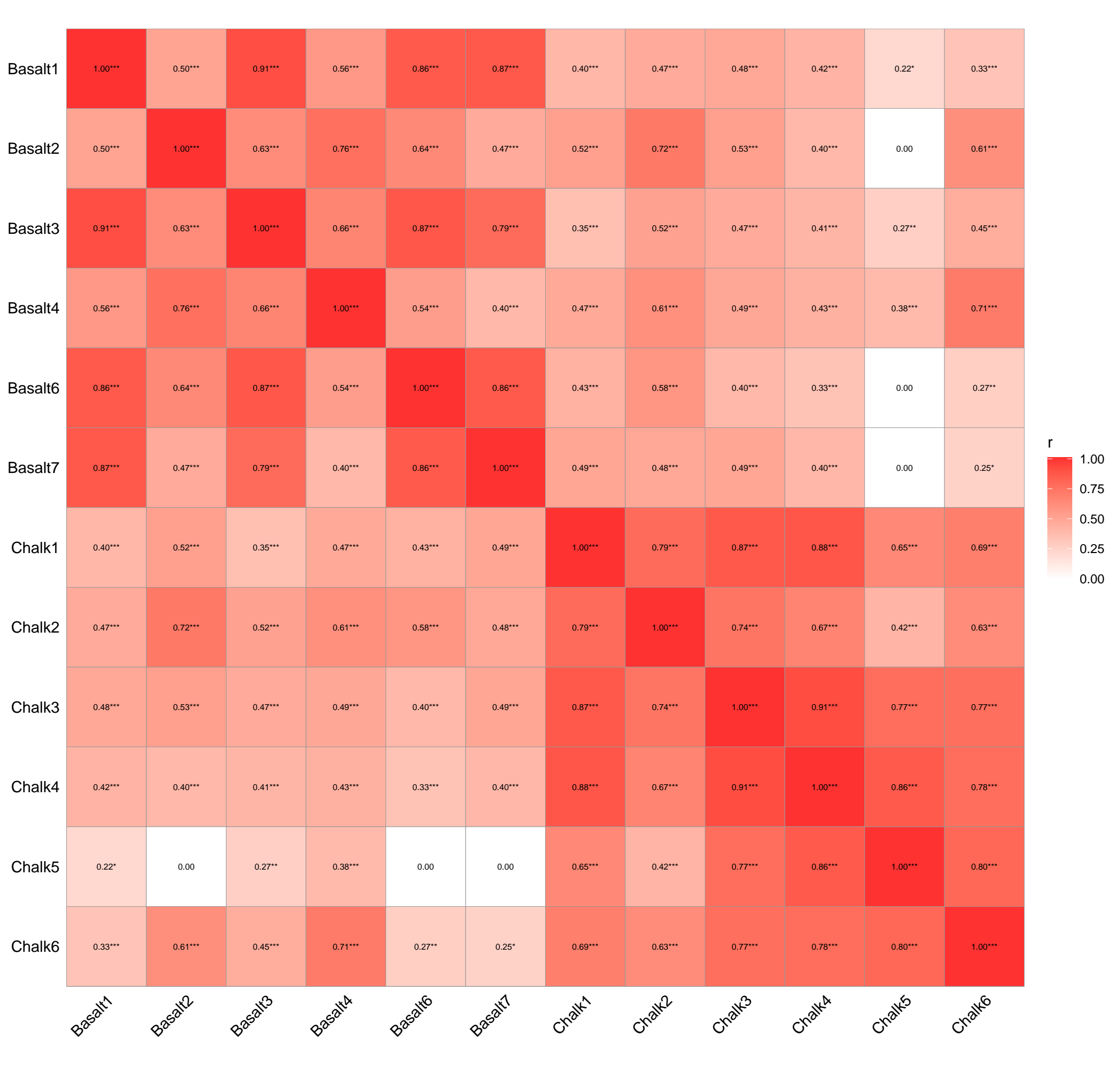

Supplement: Supplementary file 1 [file biology-11-01110-s001.zip › Supplementary Figure S1.pdf]

# *Gemmatirosa kalamazoonesis*

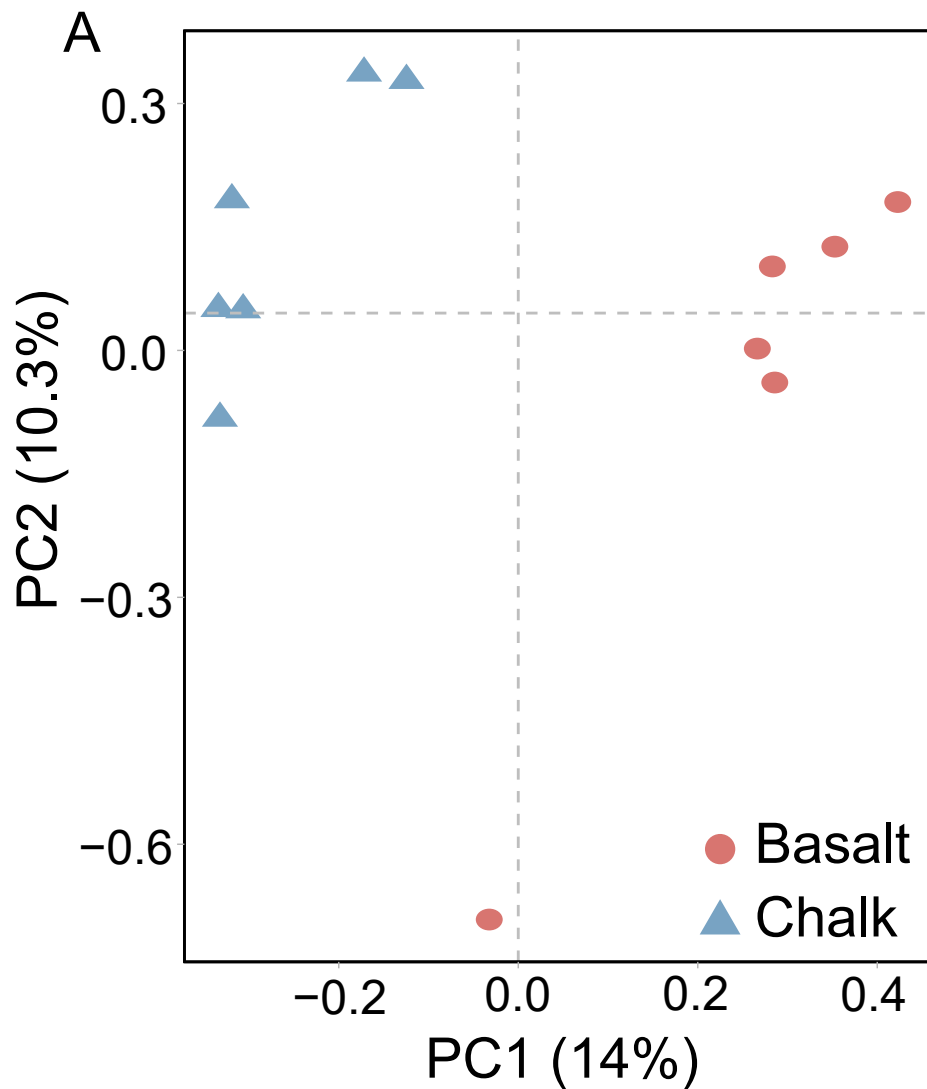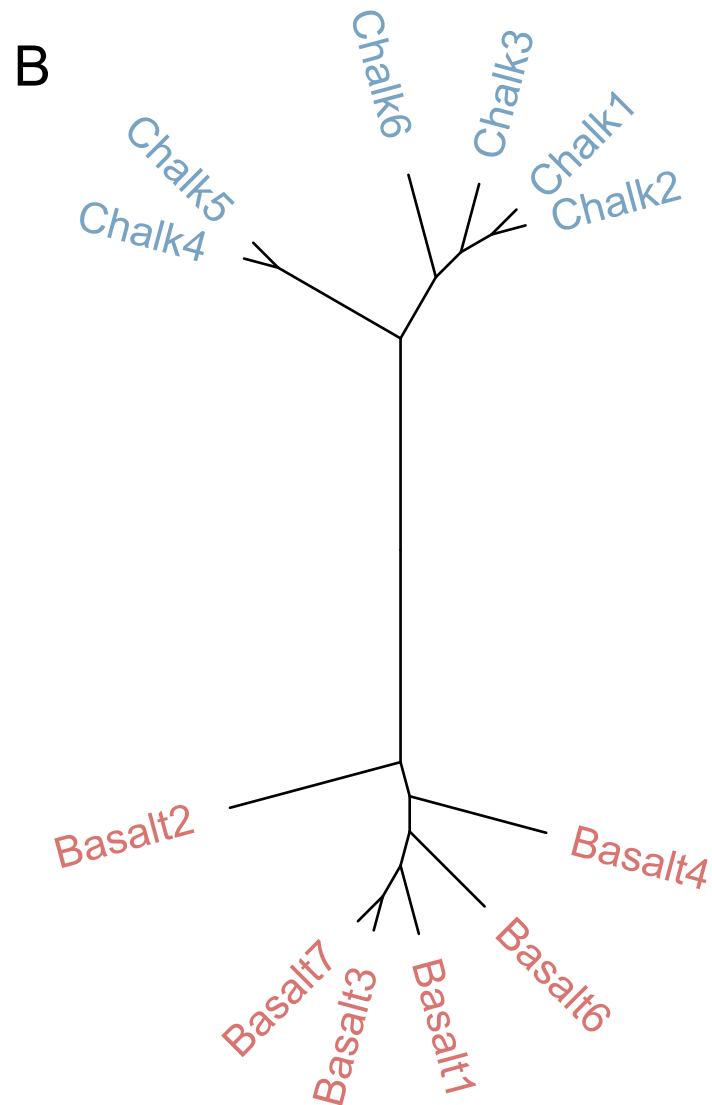

Supplement: Supplementary file 1 [file biology-11-01110-s001.zip › Supplementary Figure S10.pdf]

# *Xanthomonas euvesicatoria*

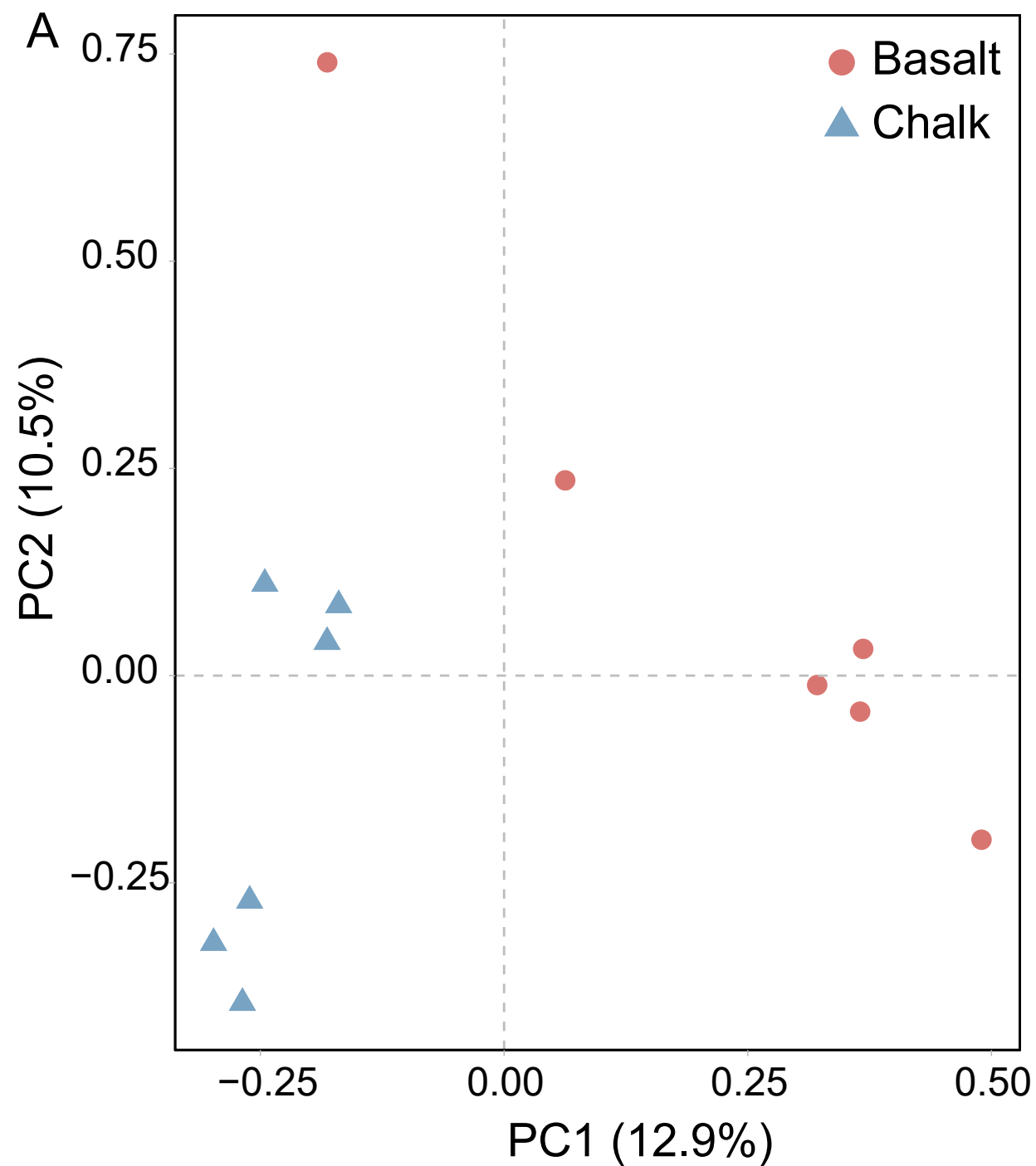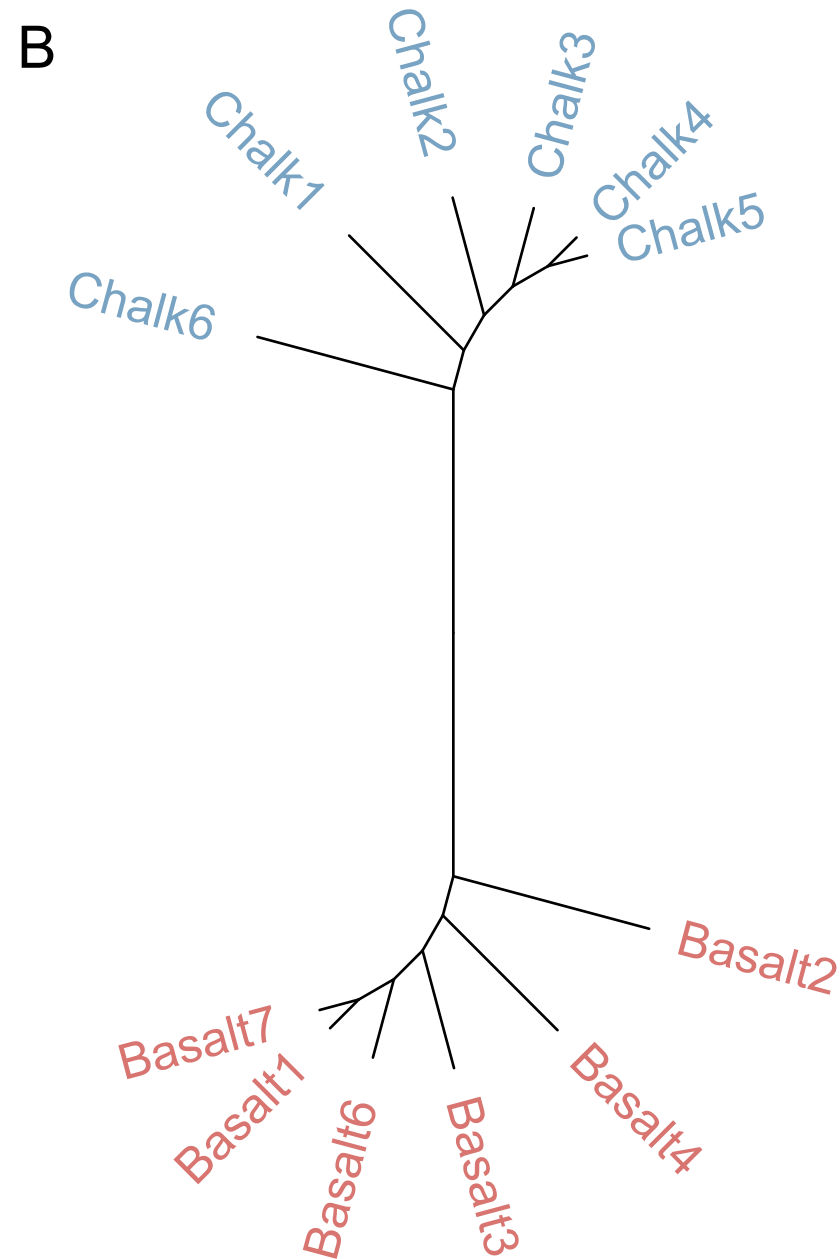

Supplement: Supplementary file 1 [file biology-11-01110-s001.zip › Supplementary Figure S11.pdf]

# *Conexibacter* sp. SYSU D00693

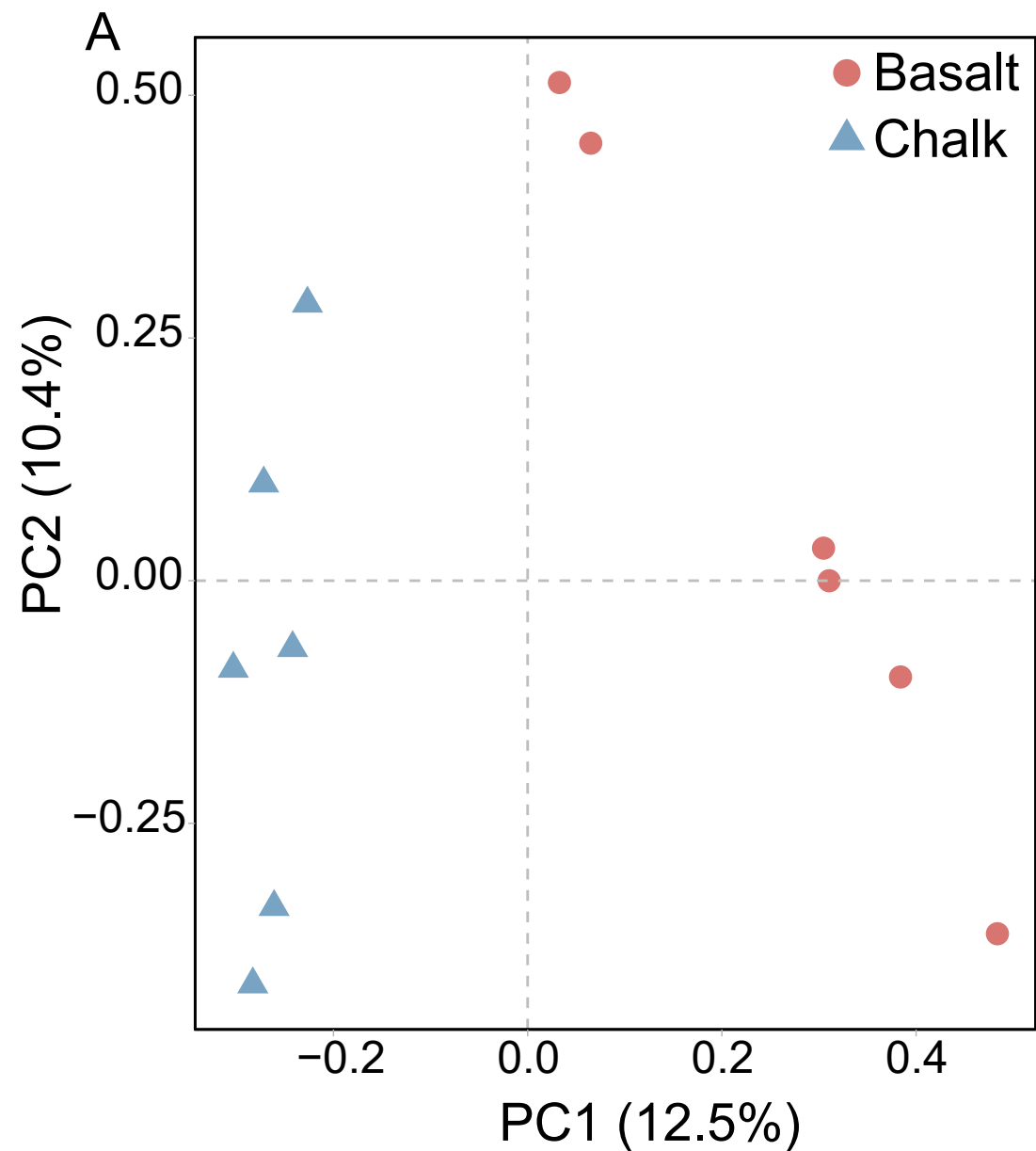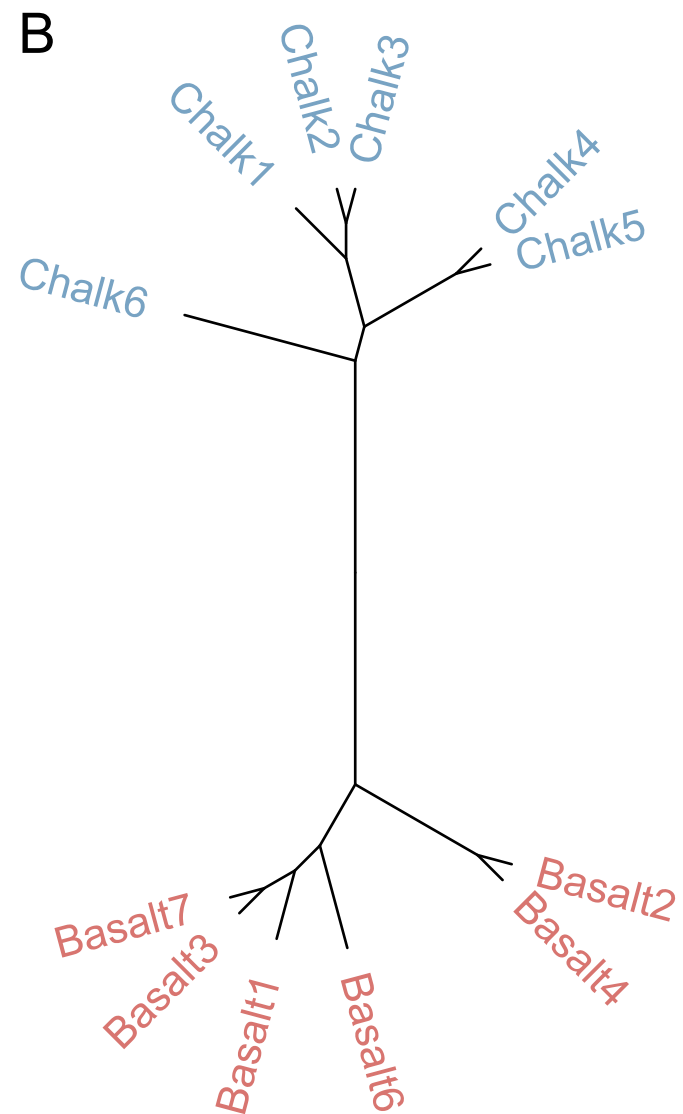

Supplement: Supplementary file 1 [file biology-11-01110-s001.zip › Supplementary Figure S12.pdf]

# *Variovorax paradoxus*

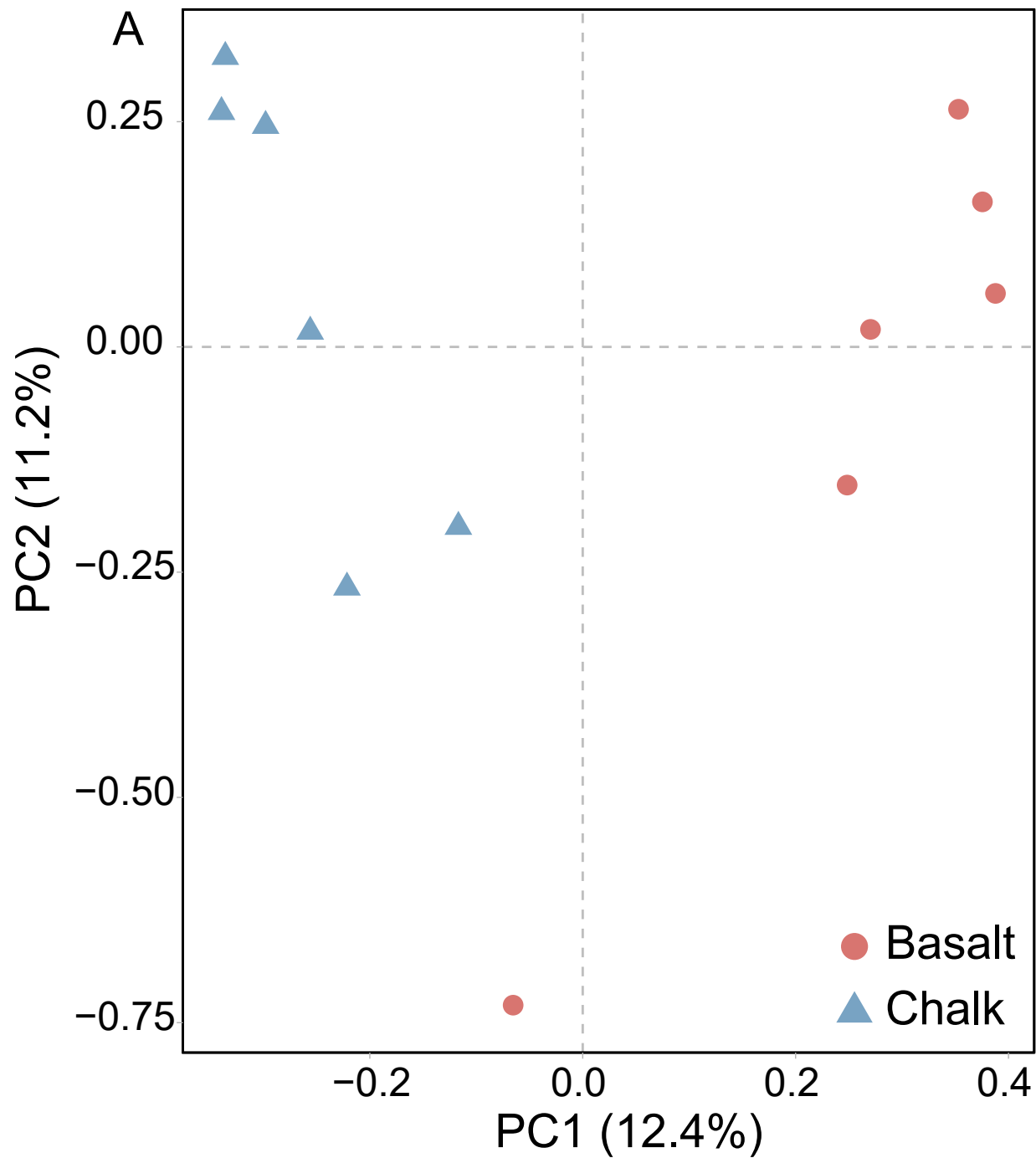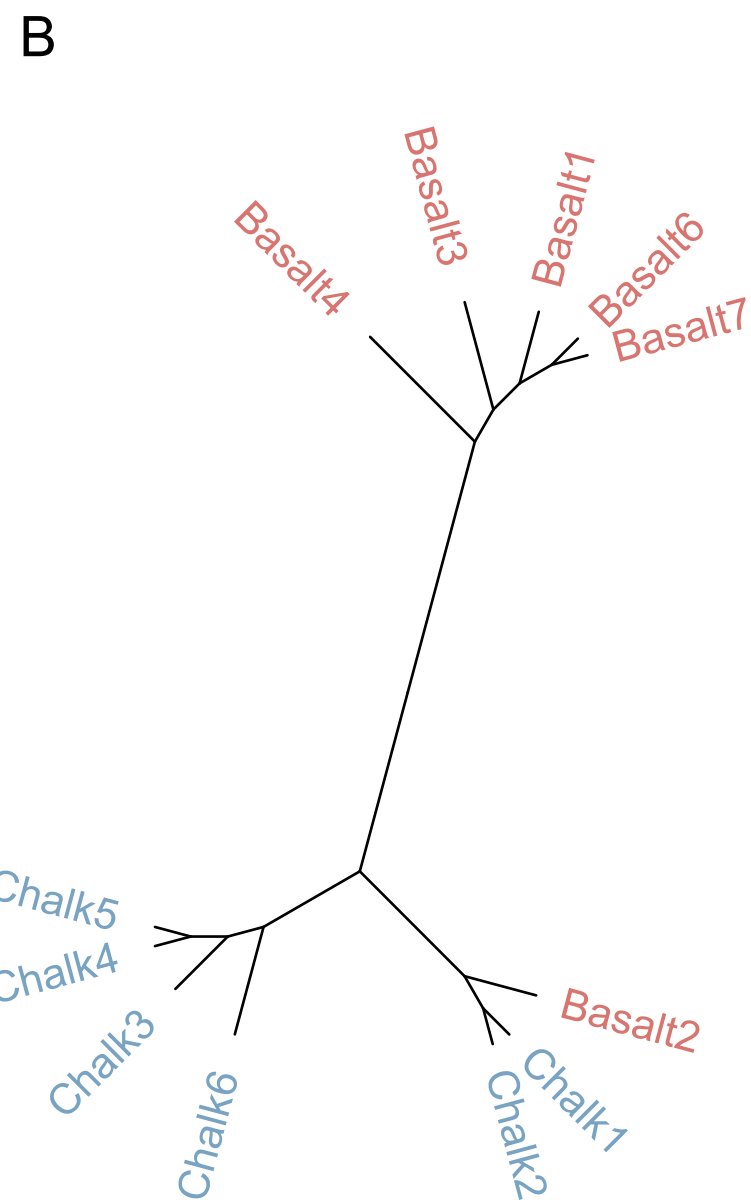

Supplement: Supplementary file 1 [file biology-11-01110-s001.zip › Supplementary Figure S13.pdf]

# *Sorangium cellulosum*

A

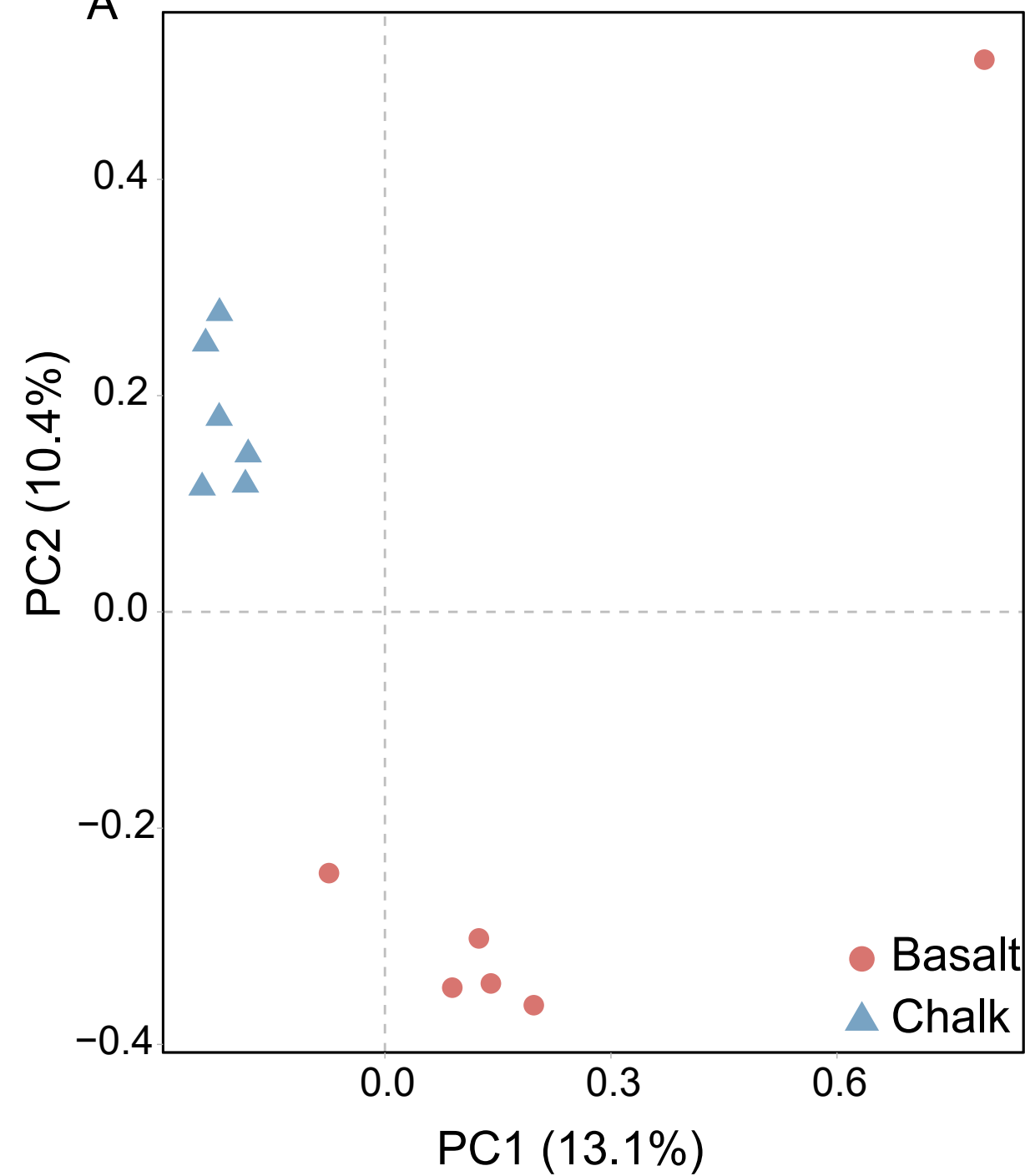

B

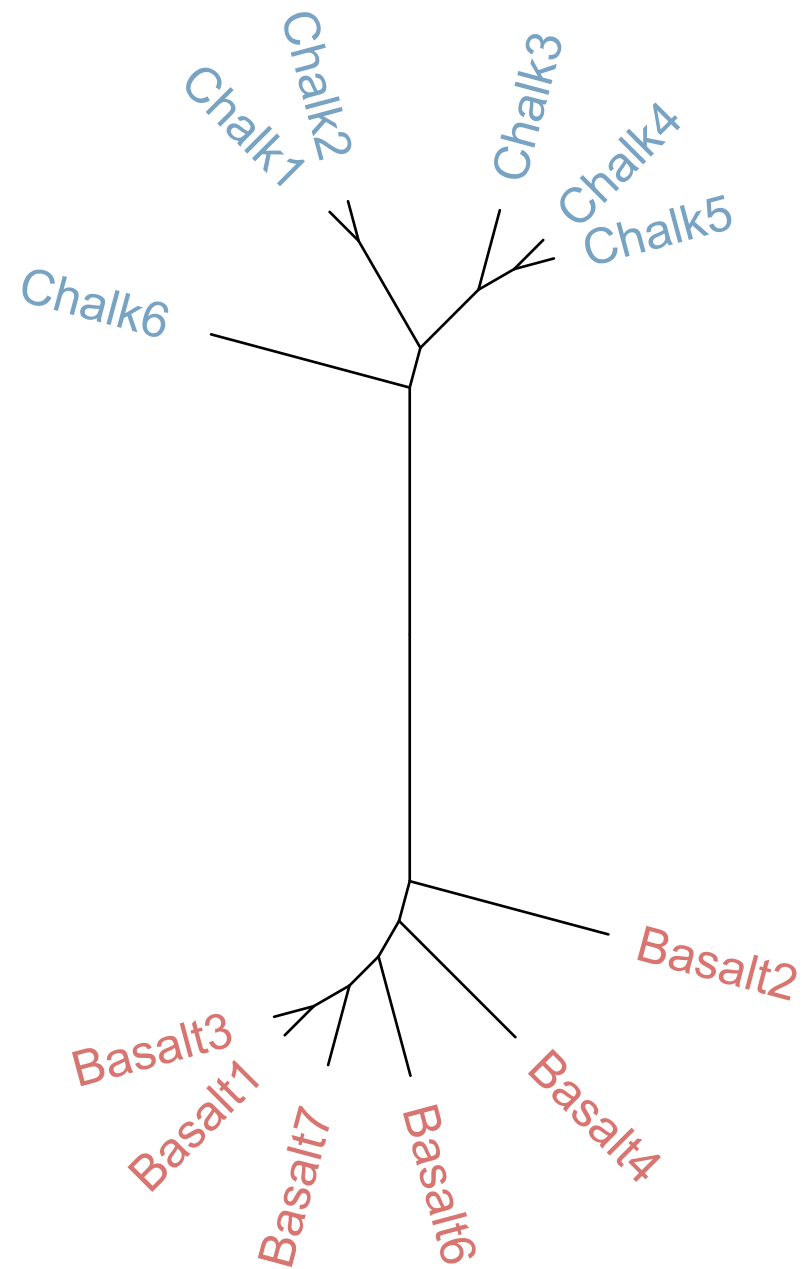

Supplement: Supplementary file 1 [file biology-11-01110-s001.zip › Supplementary Figure S14.pdf]

# *Pseudomonas aeruginosa*

A

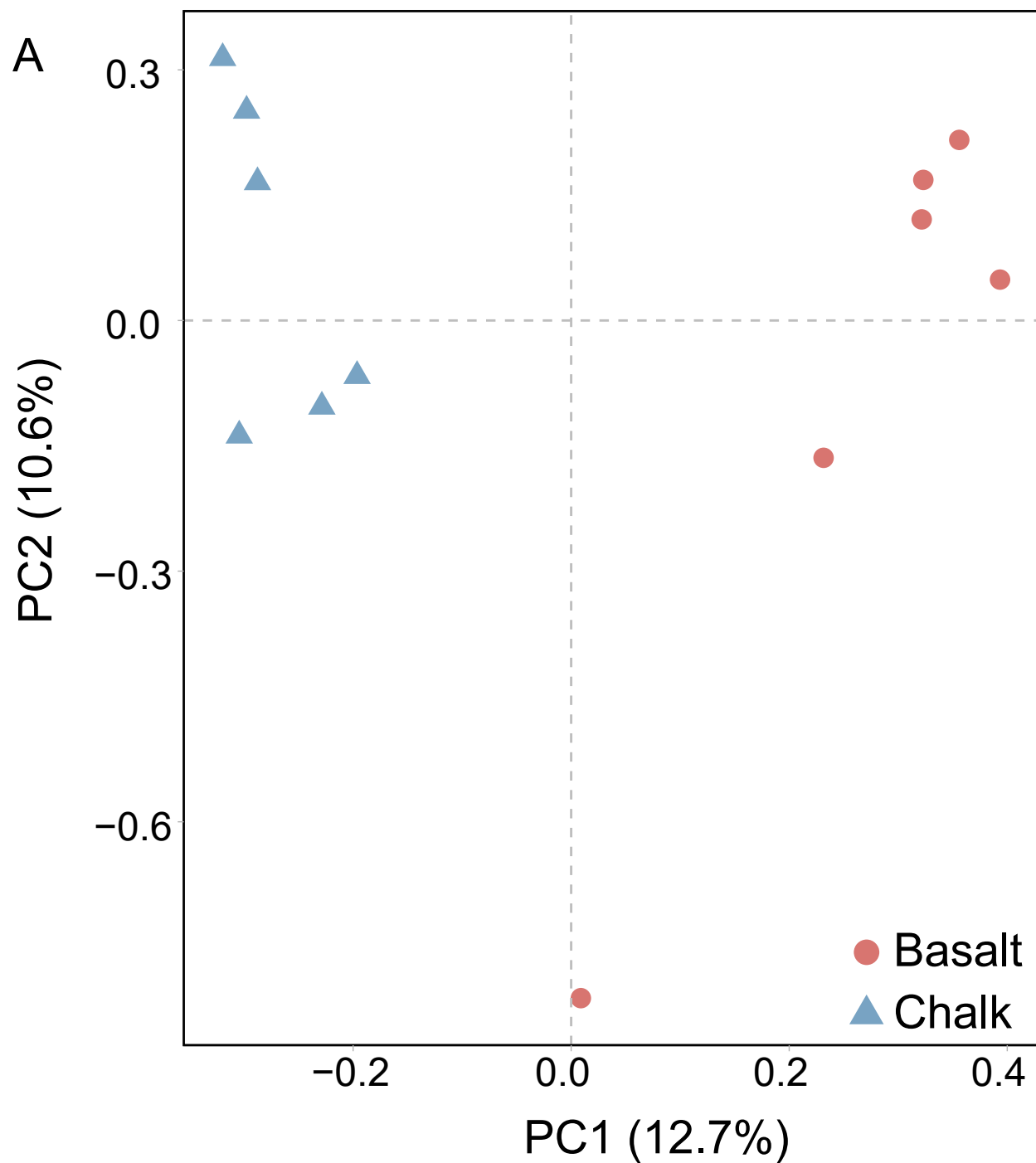

B

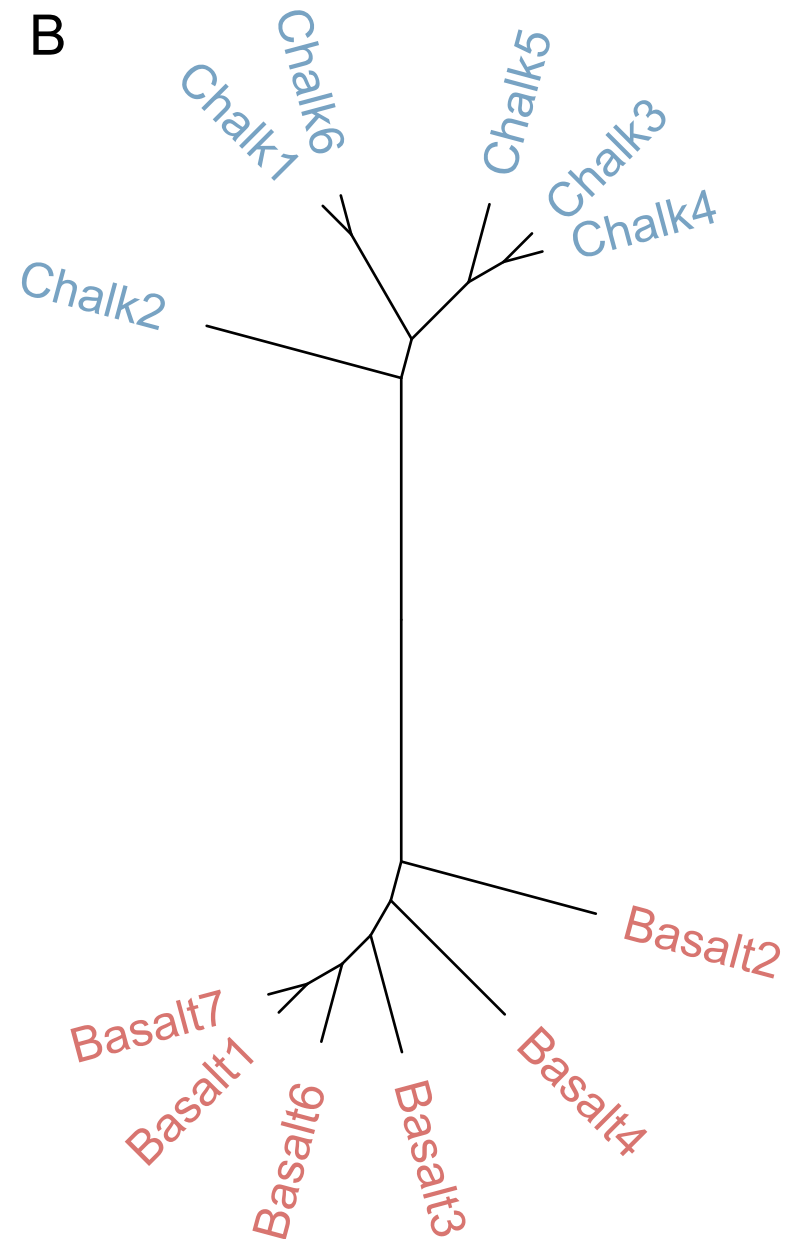

Supplement: Supplementary file 1 [file biology-11-01110-s001.zip › Supplementary Figure S15.pdf]

# *Baekduia soli*

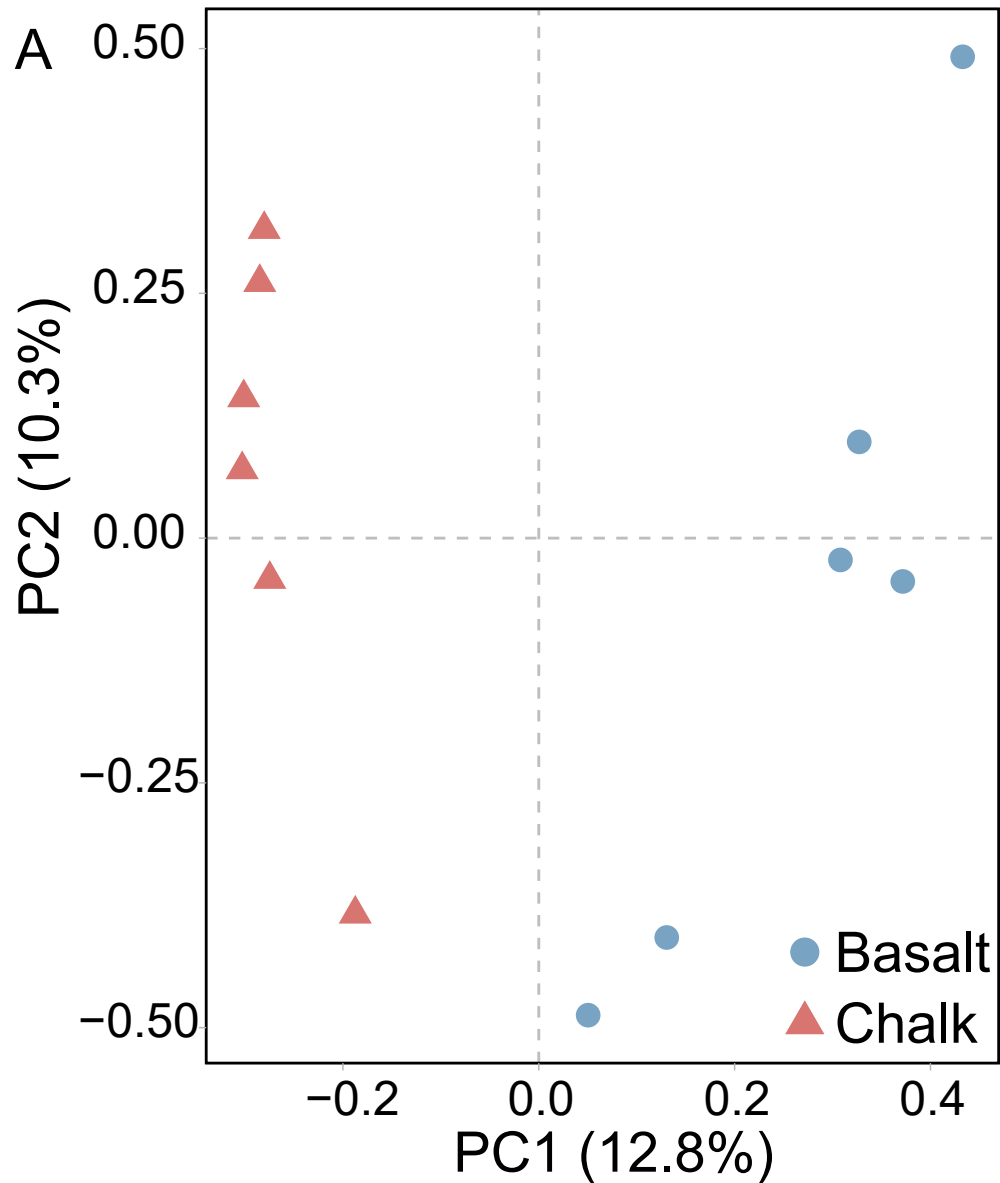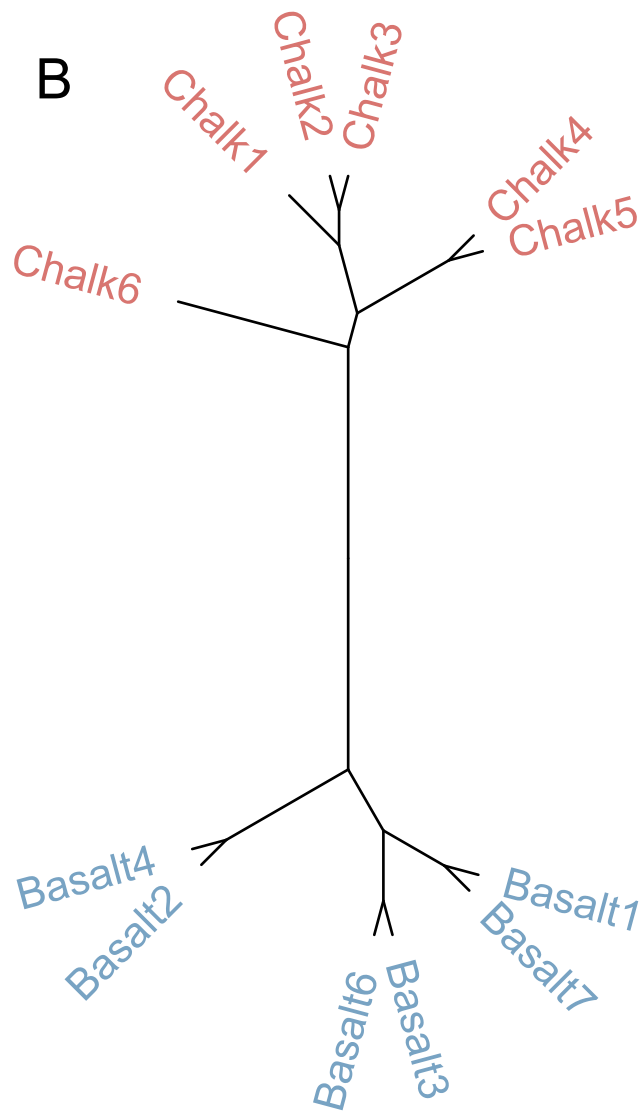

Supplement: Supplementary file 1 [file biology-11-01110-s001.zip › Supplementary Figure S16.pdf]

*Luteitalea pratensis*

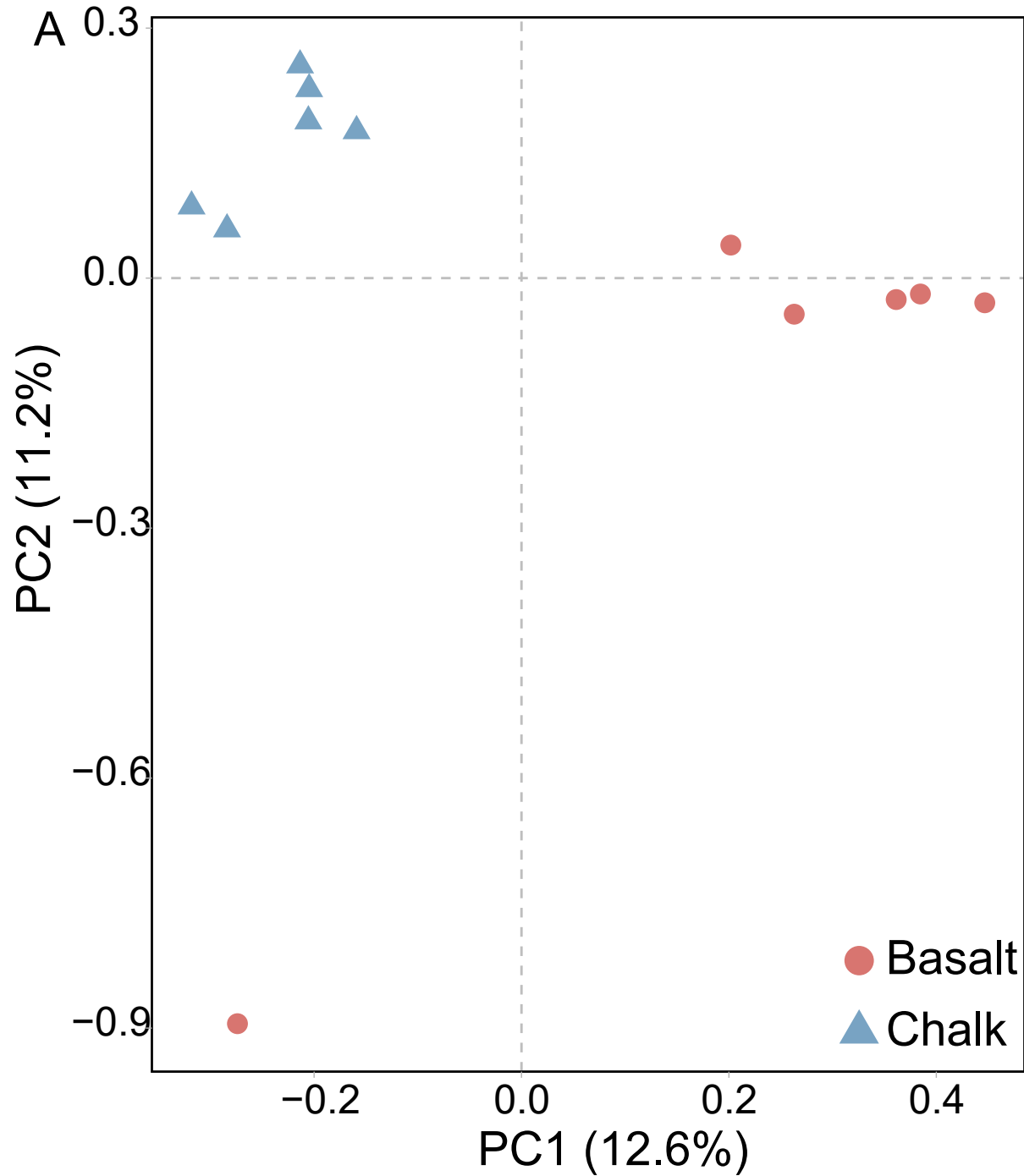

B

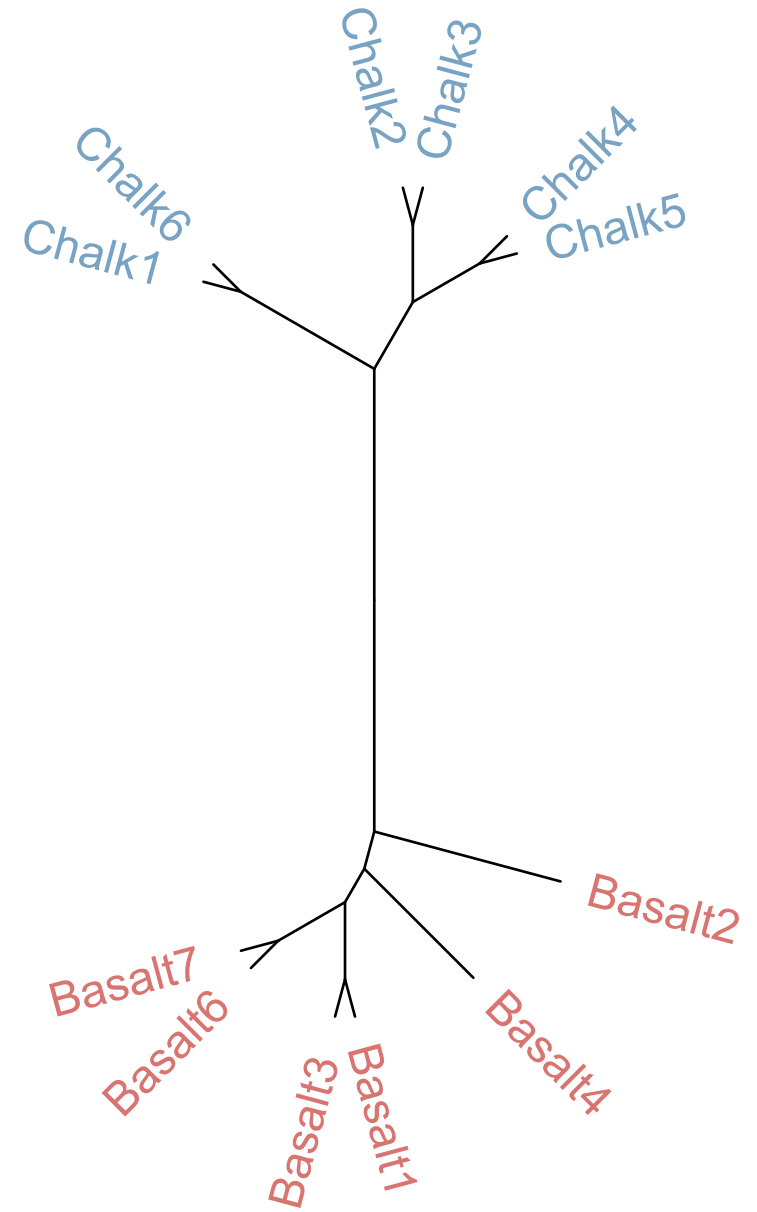

Supplement: Supplementary file 1 [file biology-11-01110-s001.zip › Supplementary Figure S17.pdf]

*Bradyrhizobium diazoefficiens*

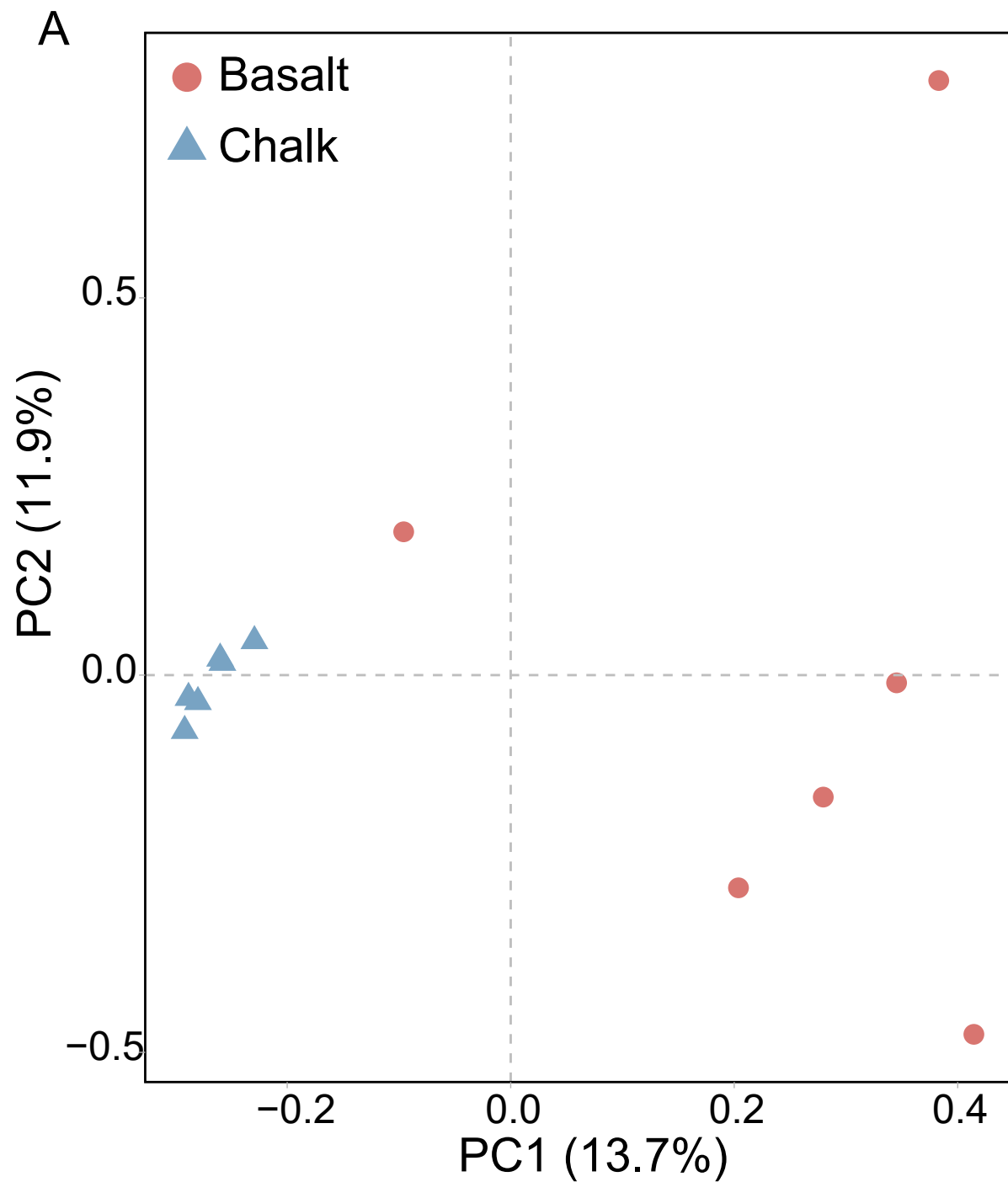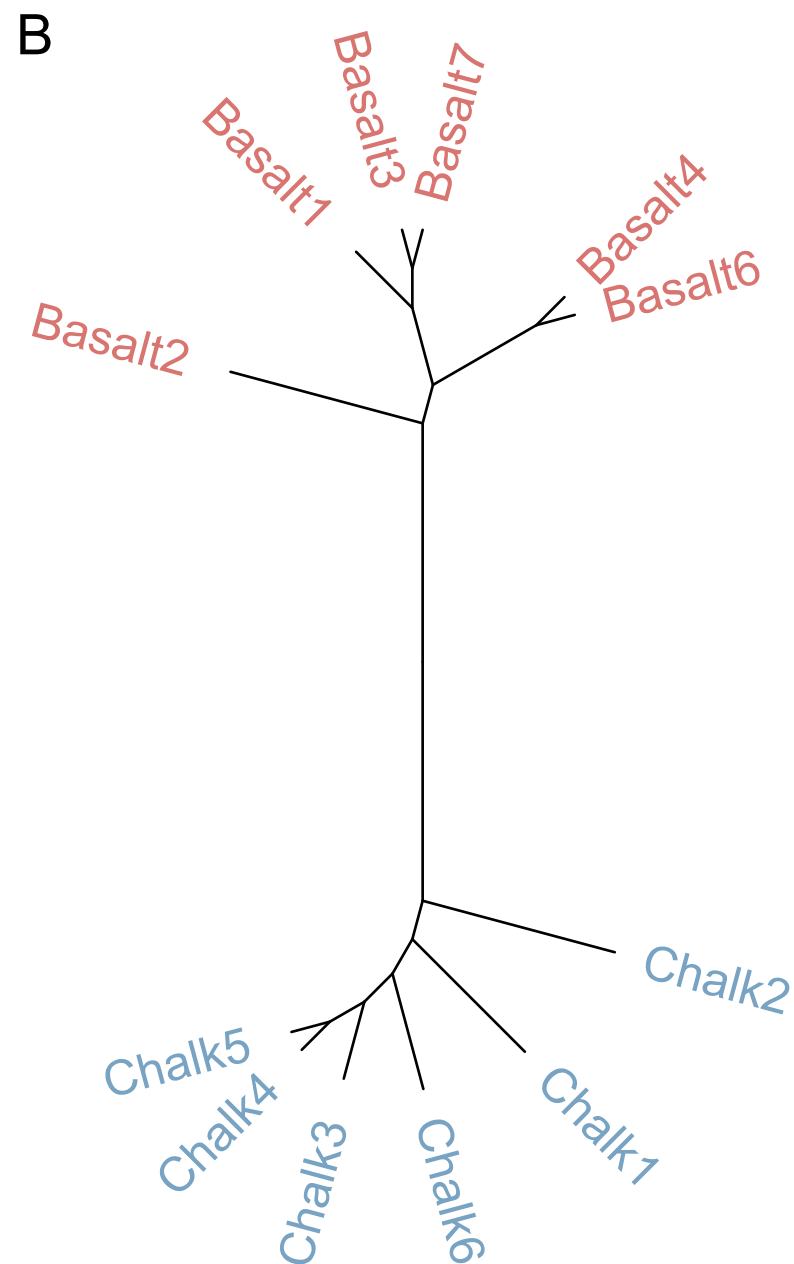

Supplement: Supplementary file 1 [file biology-11-01110-s001.zip › Supplementary Figure S18.pdf]

*Bradyrhizobium sp. CCBAU 051011*

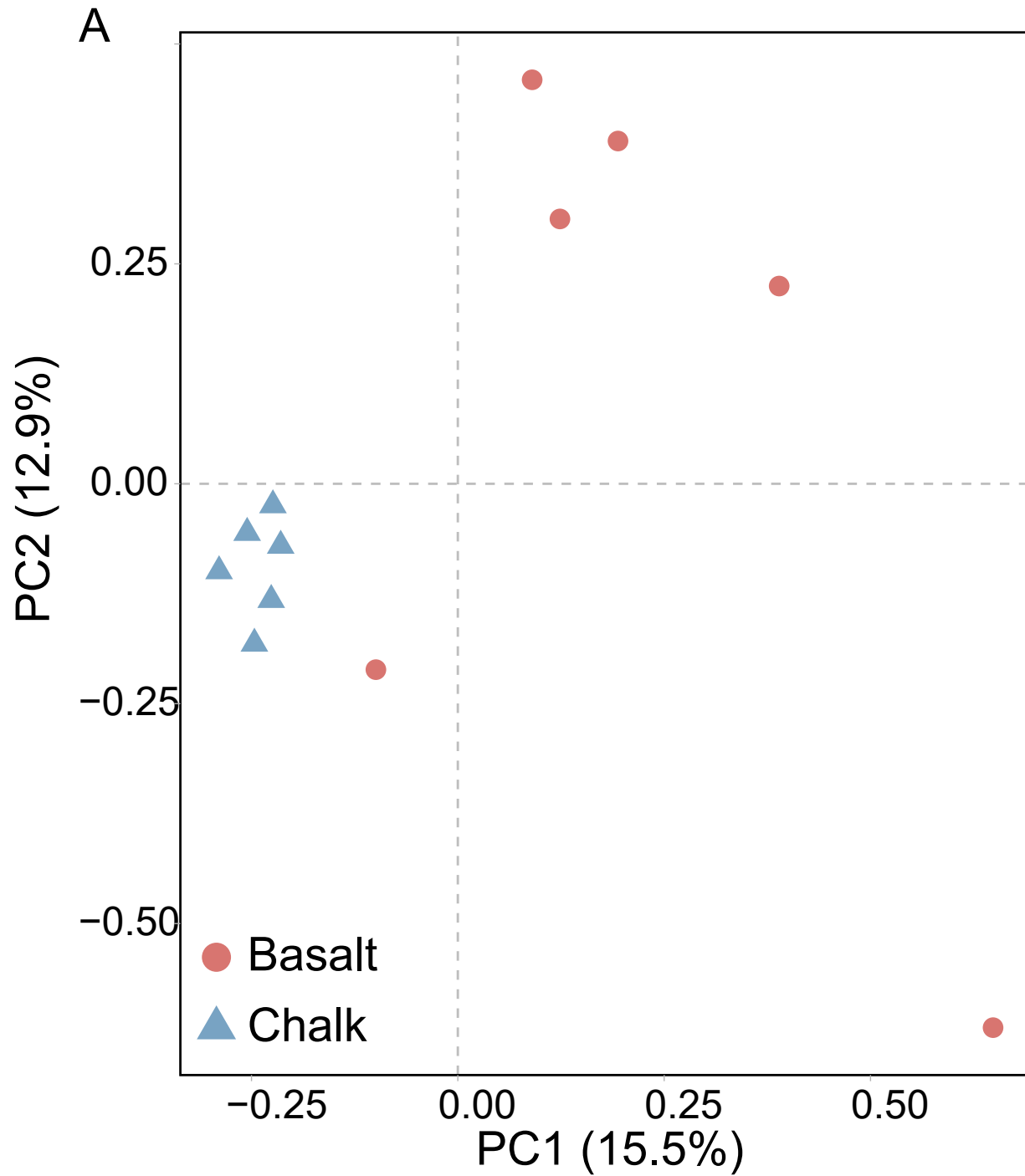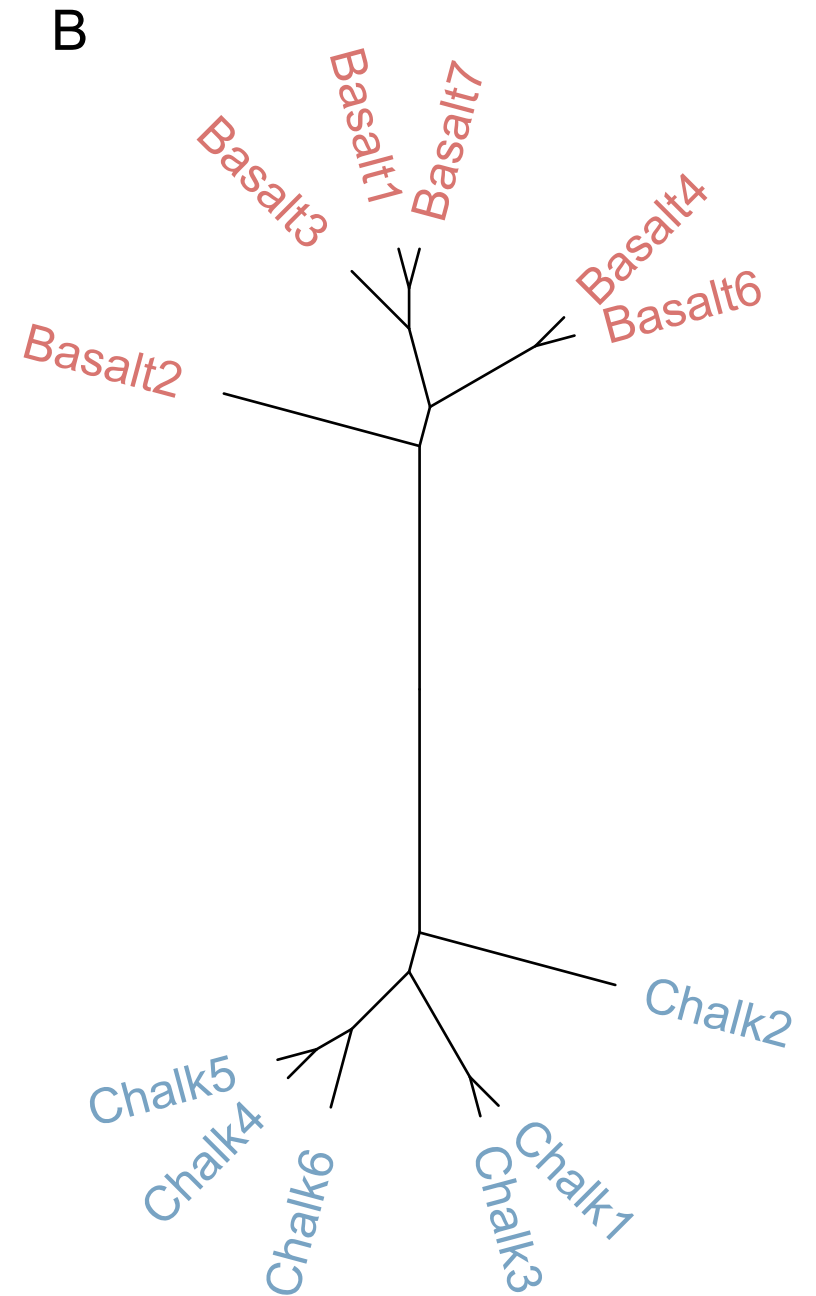

Supplement: Supplementary file 1 [file biology-11-01110-s001.zip › Supplementary Figure S19.pdf]

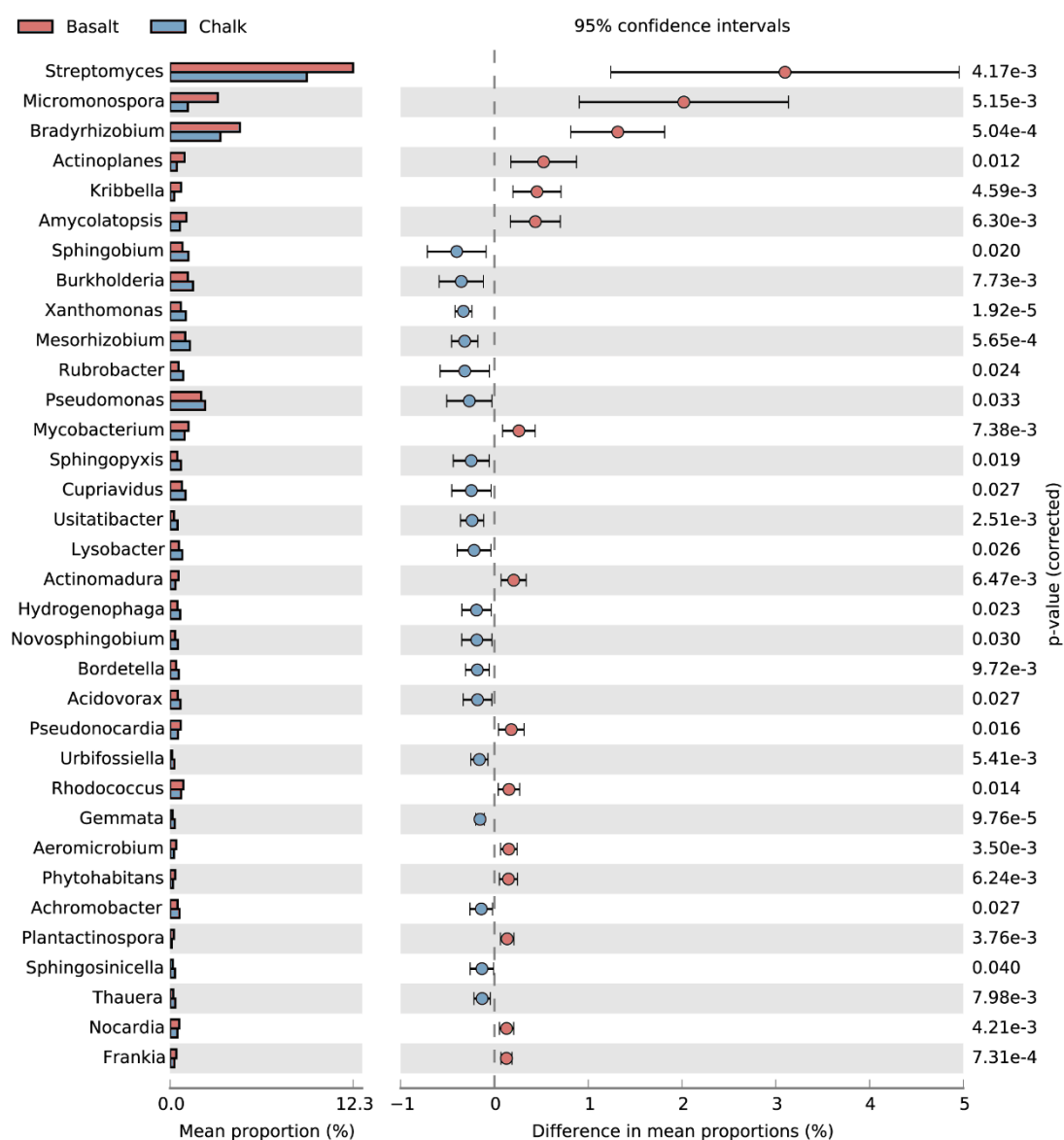

**Figure S1.** Level of significance for relative abundances of all Genera in soil samples.

Supplement: Supplementary file 1 [file biology-11-01110-s001.zip › Supplementary Figure S2.pdf]

*Bradyrhizobium icense*

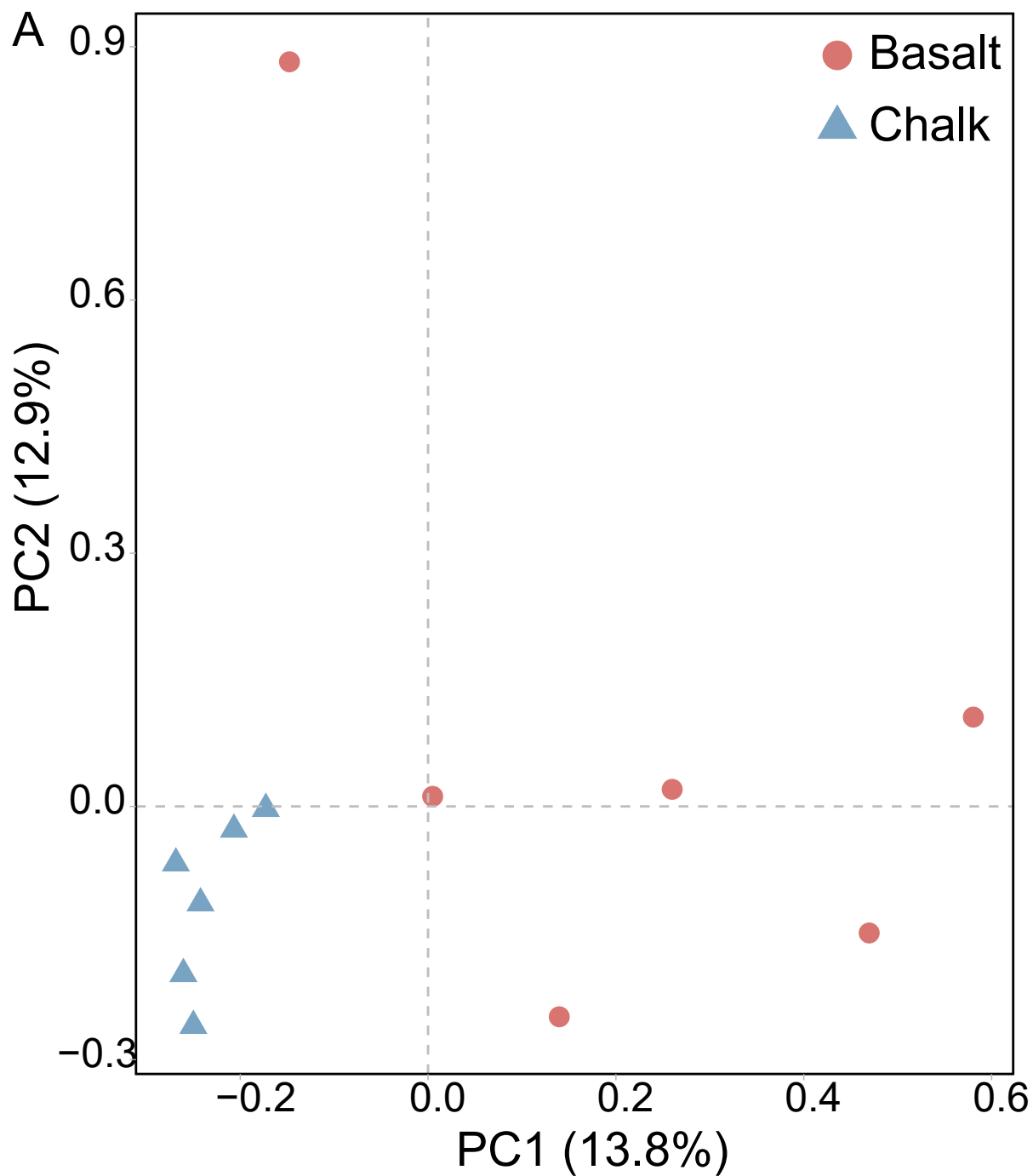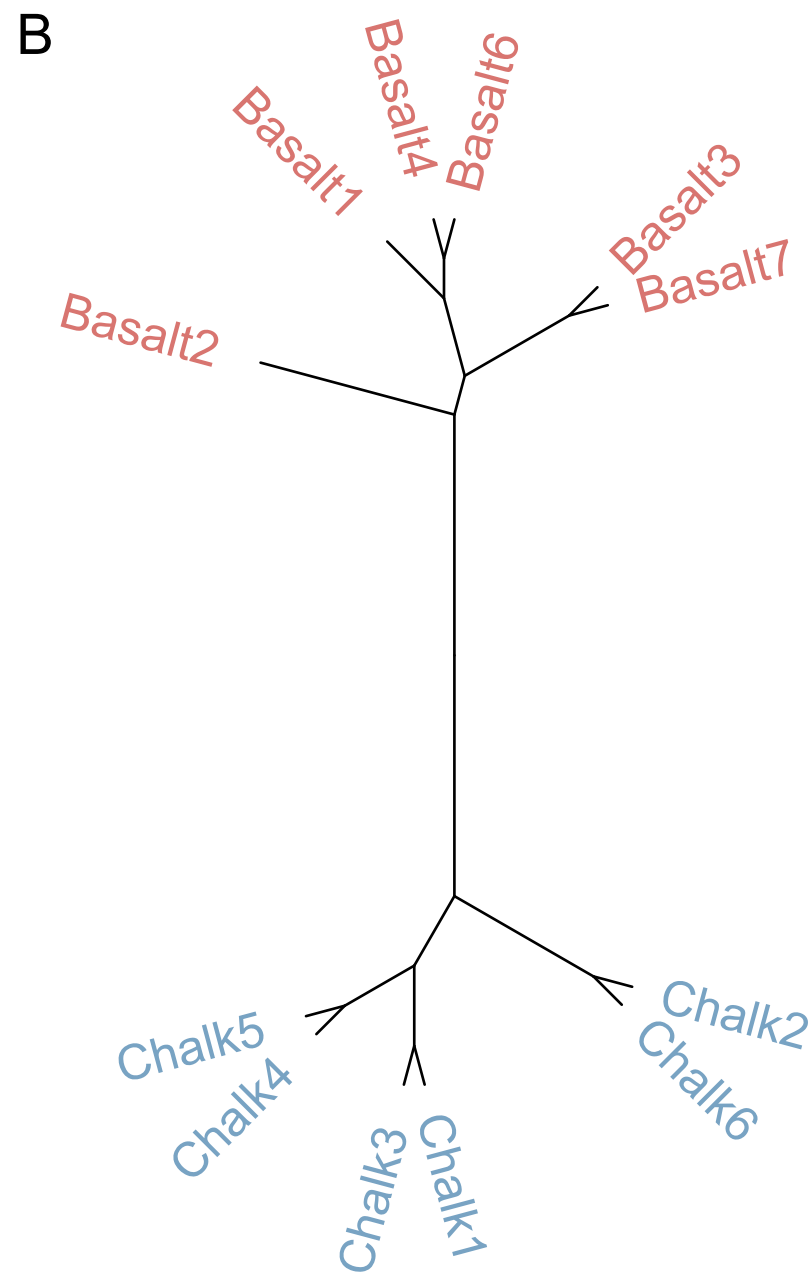

Supplement: Supplementary file 1 [file biology-11-01110-s001.zip › Supplementary Figure S20.pdf]

*Bradyrhizobium lablabi*

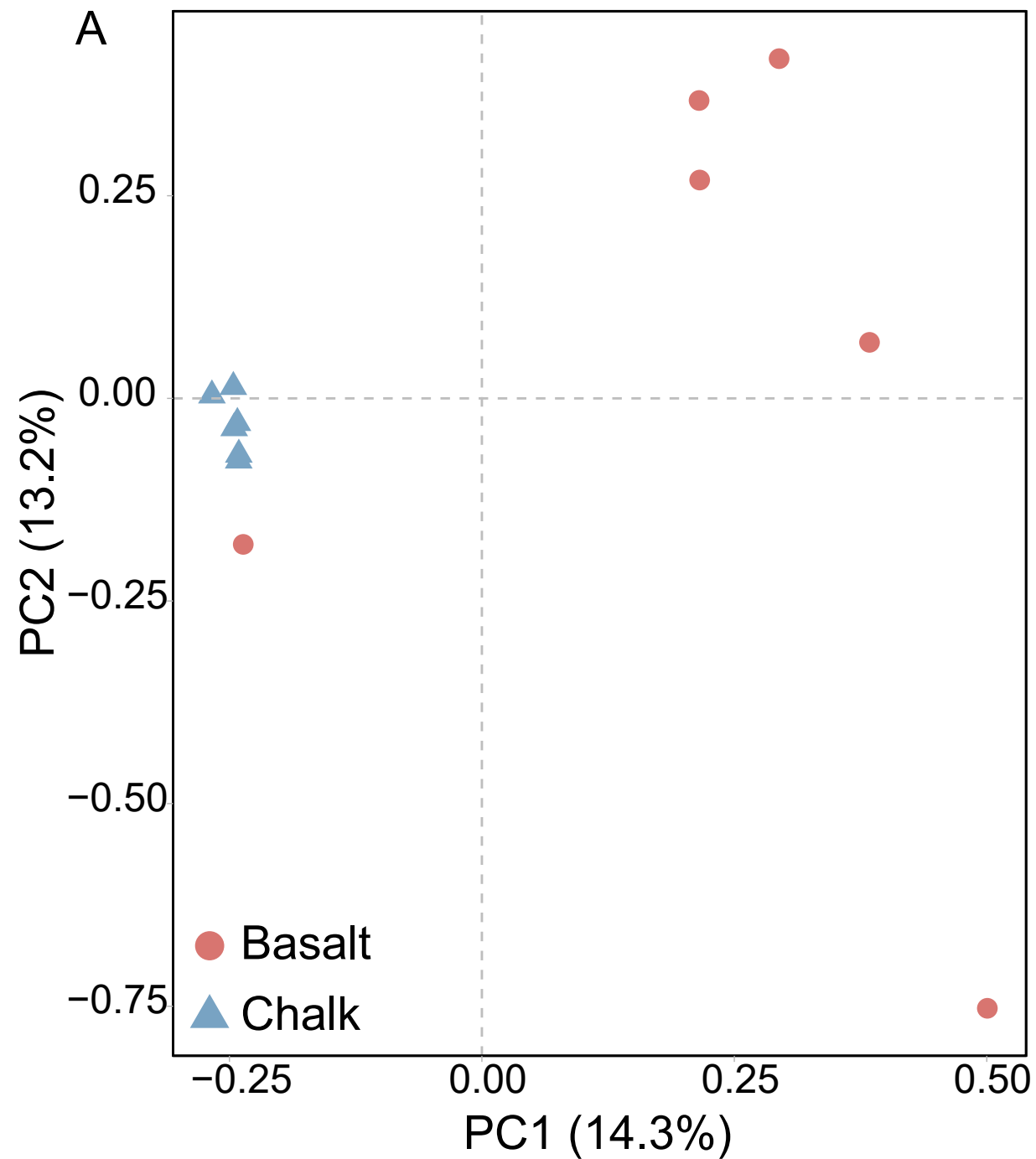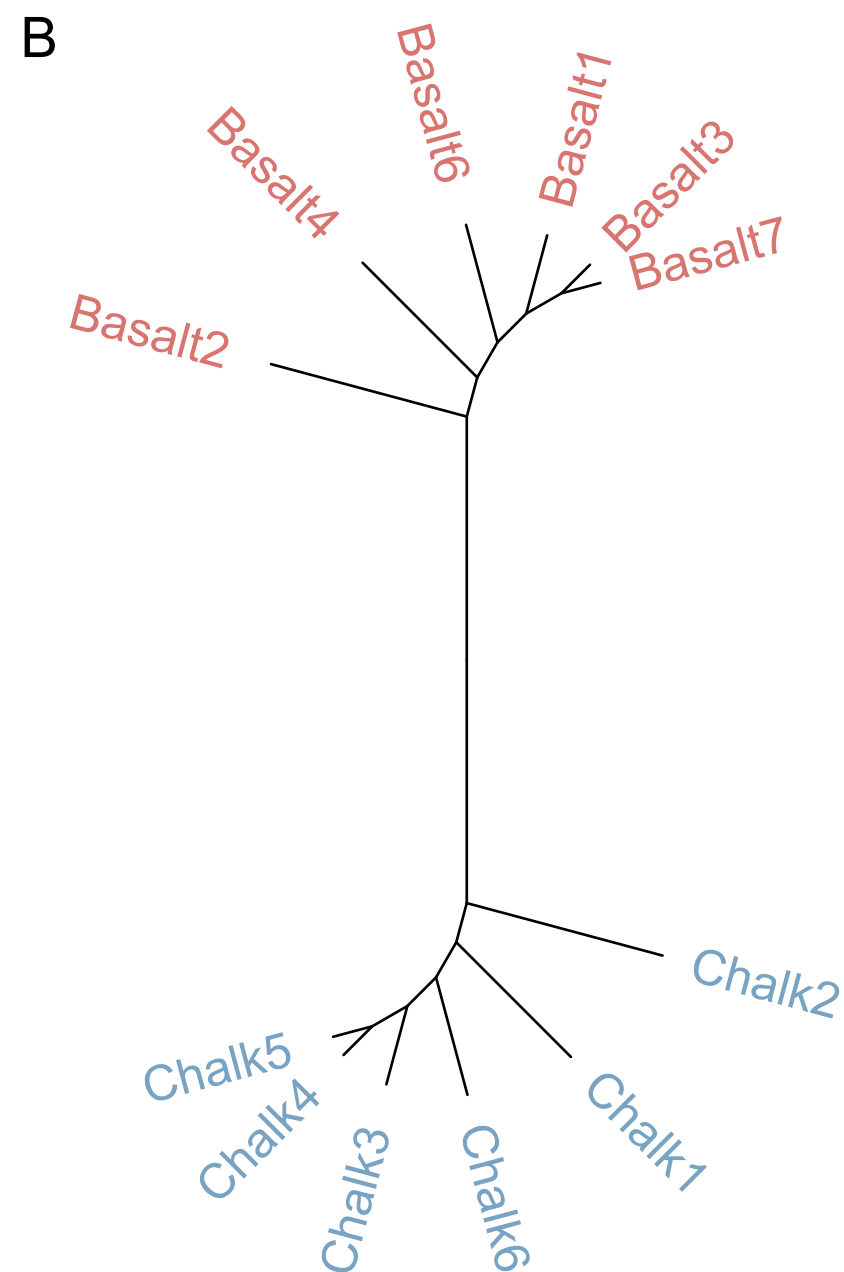

Supplement: Supplementary file 1 [file biology-11-01110-s001.zip › Supplementary Figure S21.pdf]

*Bradyrhizobium erythrophlei*

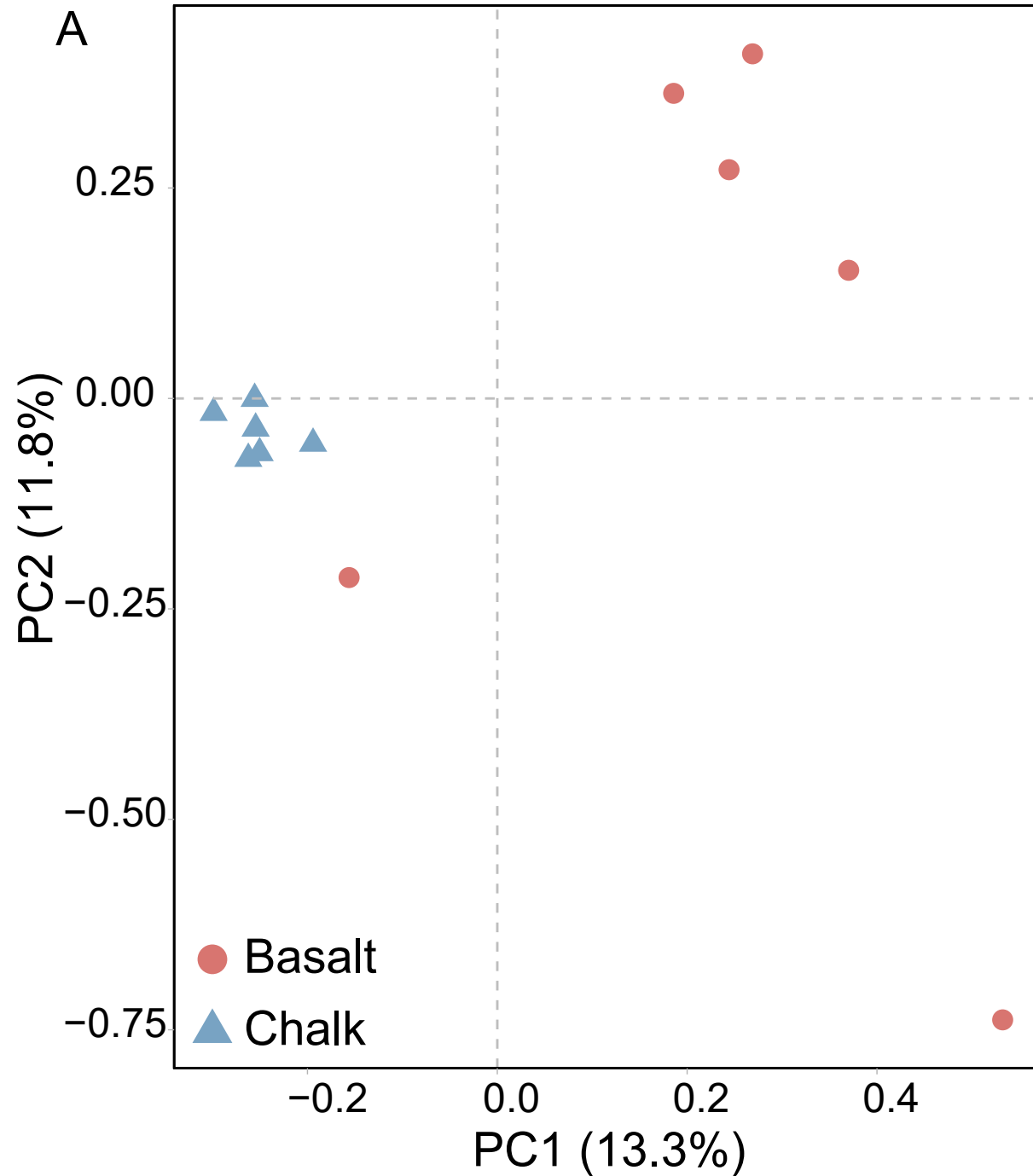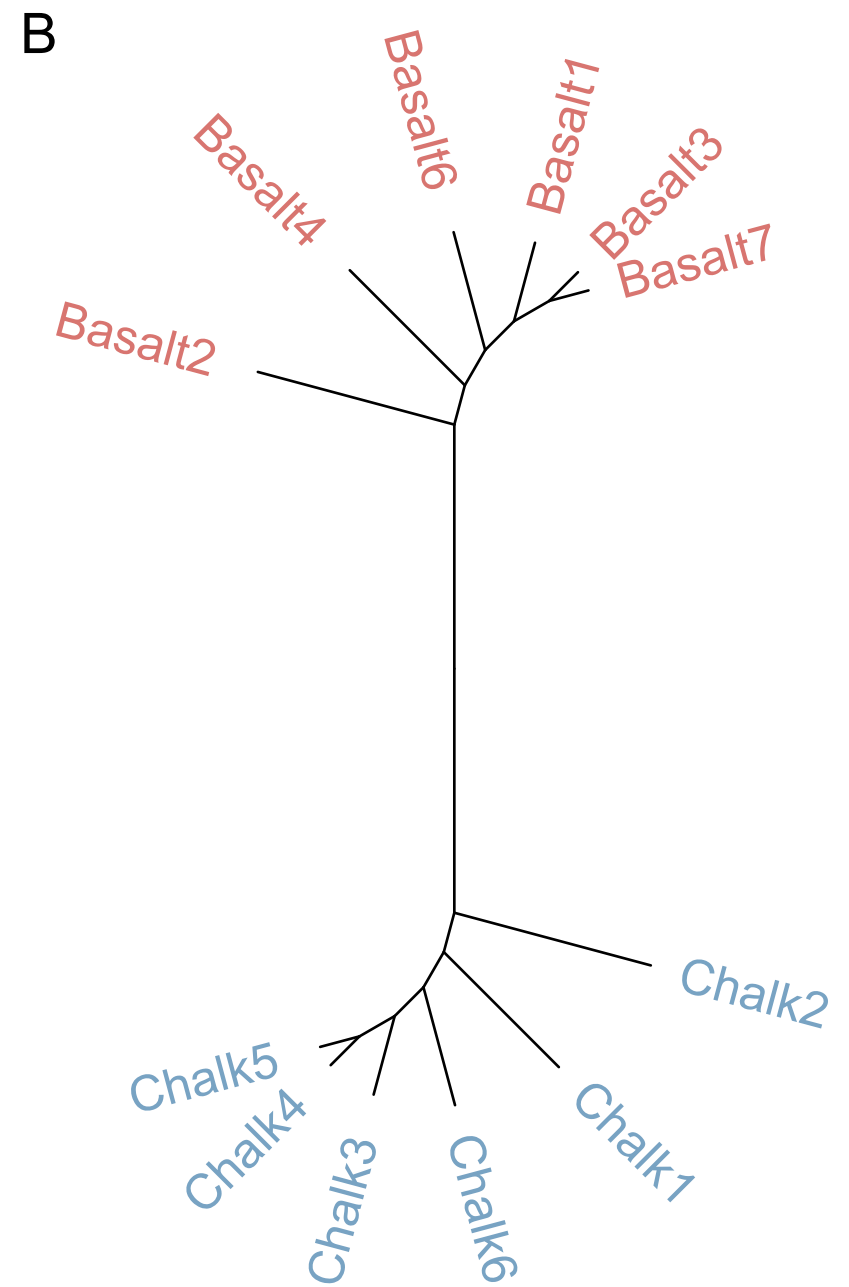

Supplement: Supplementary file 1 [file biology-11-01110-s001.zip › Supplementary Figure S22.pdf]

*Ramlibacter tataouinensis*

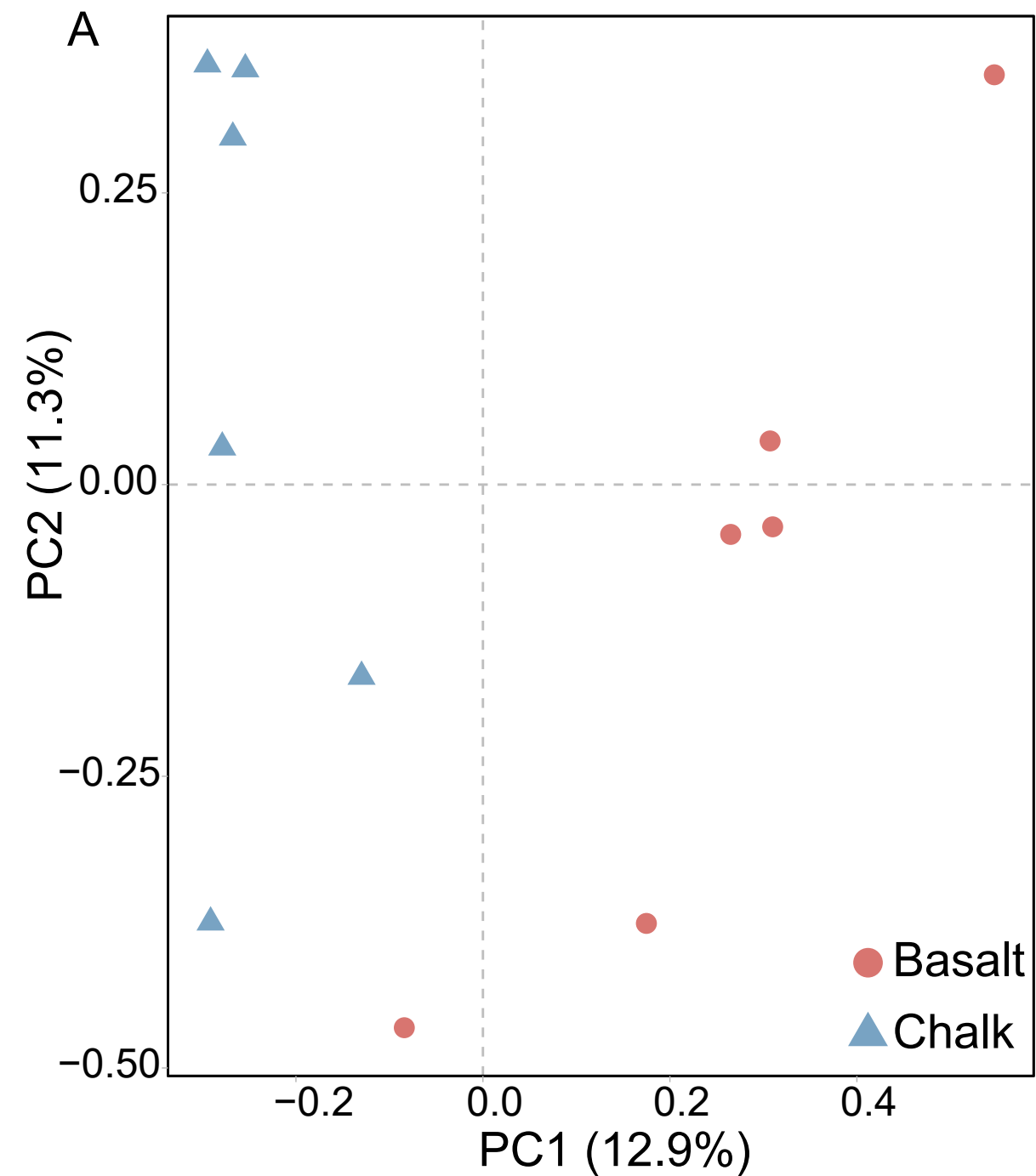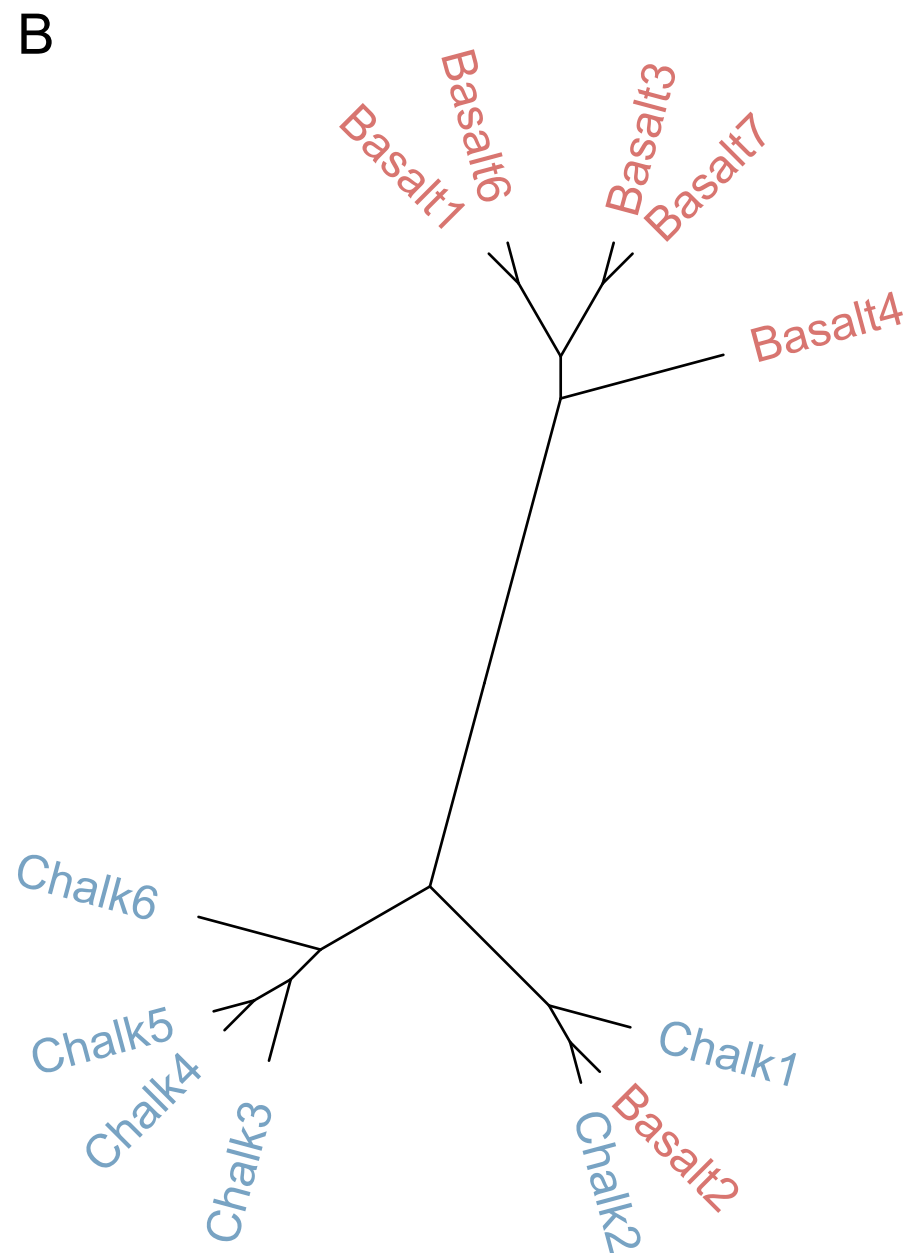

Supplement: Supplementary file 1 [file biology-11-01110-s001.zip › Supplementary Figure S23.pdf]

*Bradyrhizobium paxllaeri*

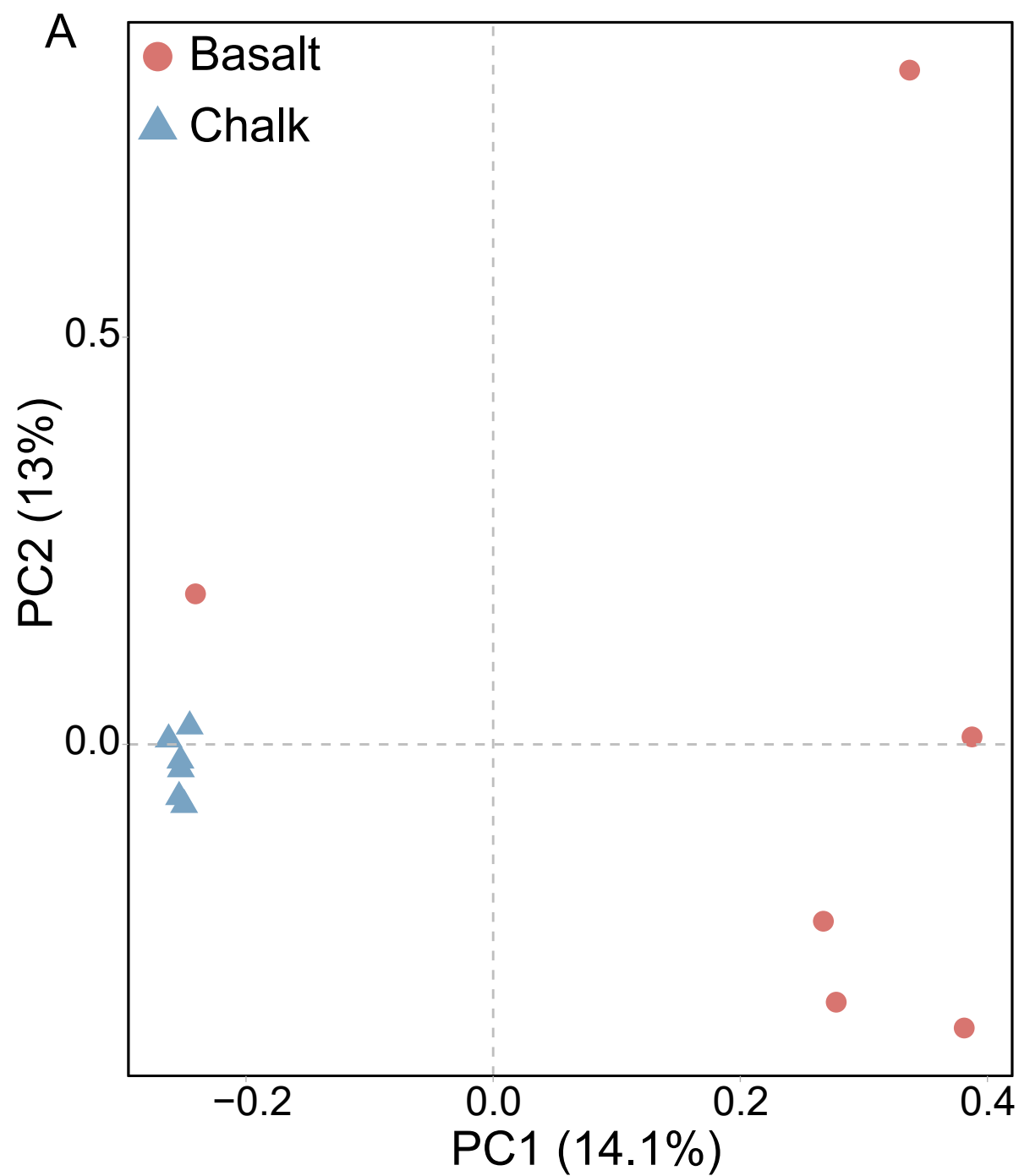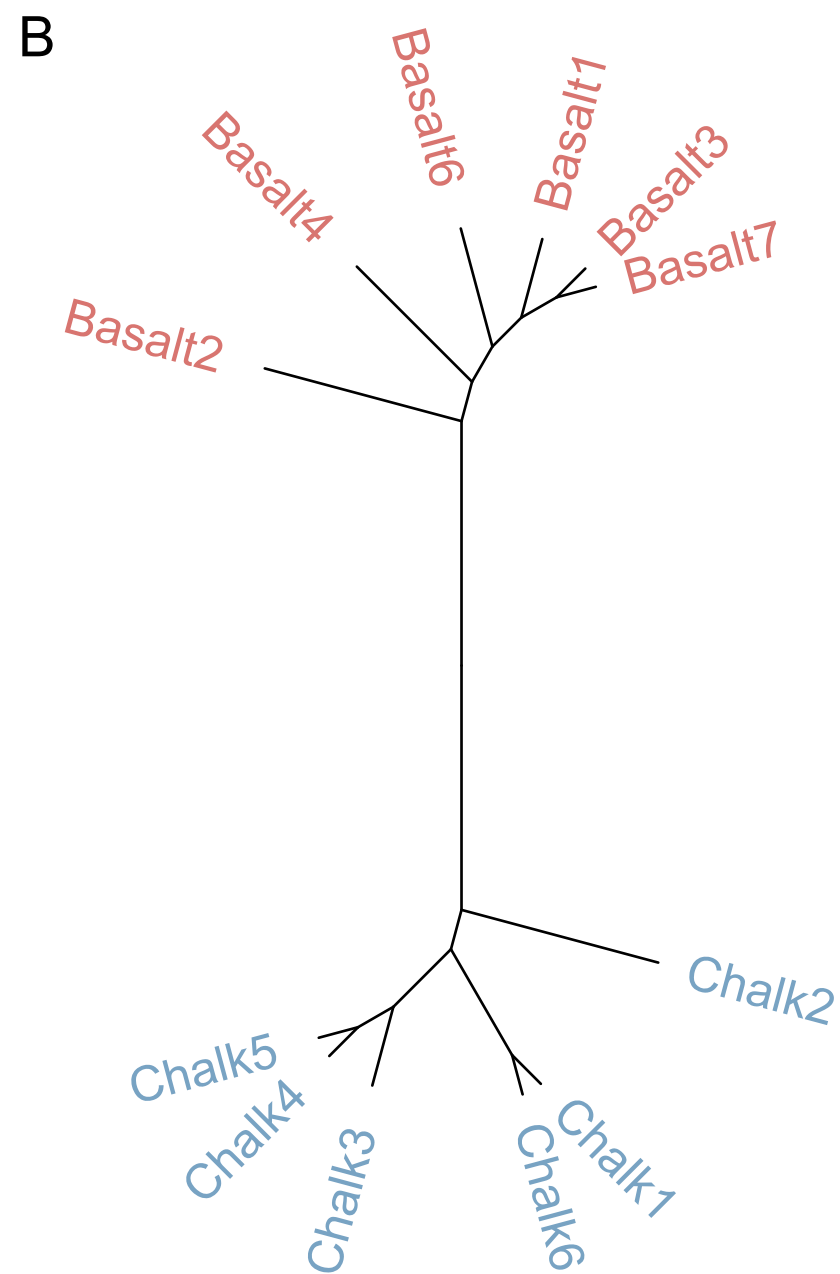

Supplement: Supplementary file 1 [file biology-11-01110-s001.zip › Supplementary Figure S24.pdf]

*Kribbella flavida*

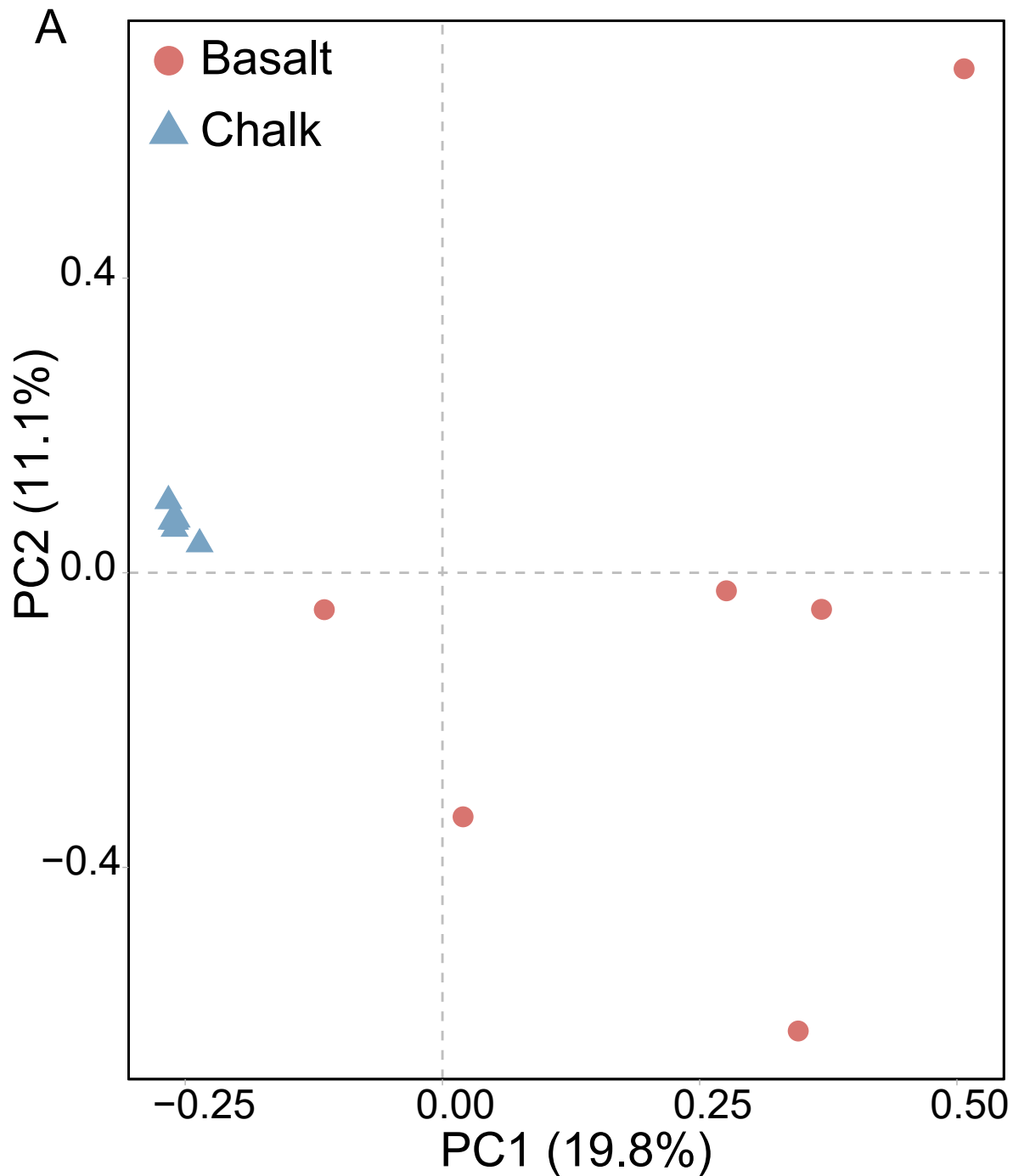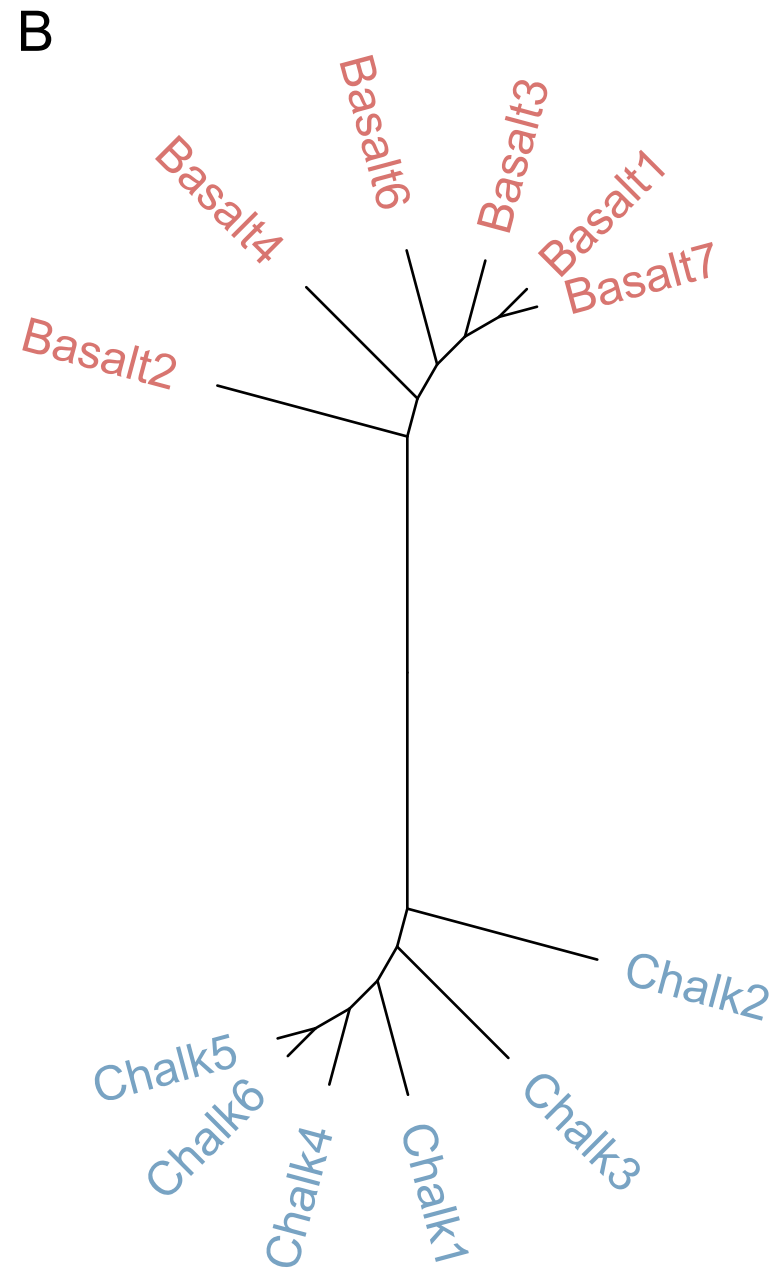

Supplement: Supplementary file 1 [file biology-11-01110-s001.zip › Supplementary Figure S25.pdf]

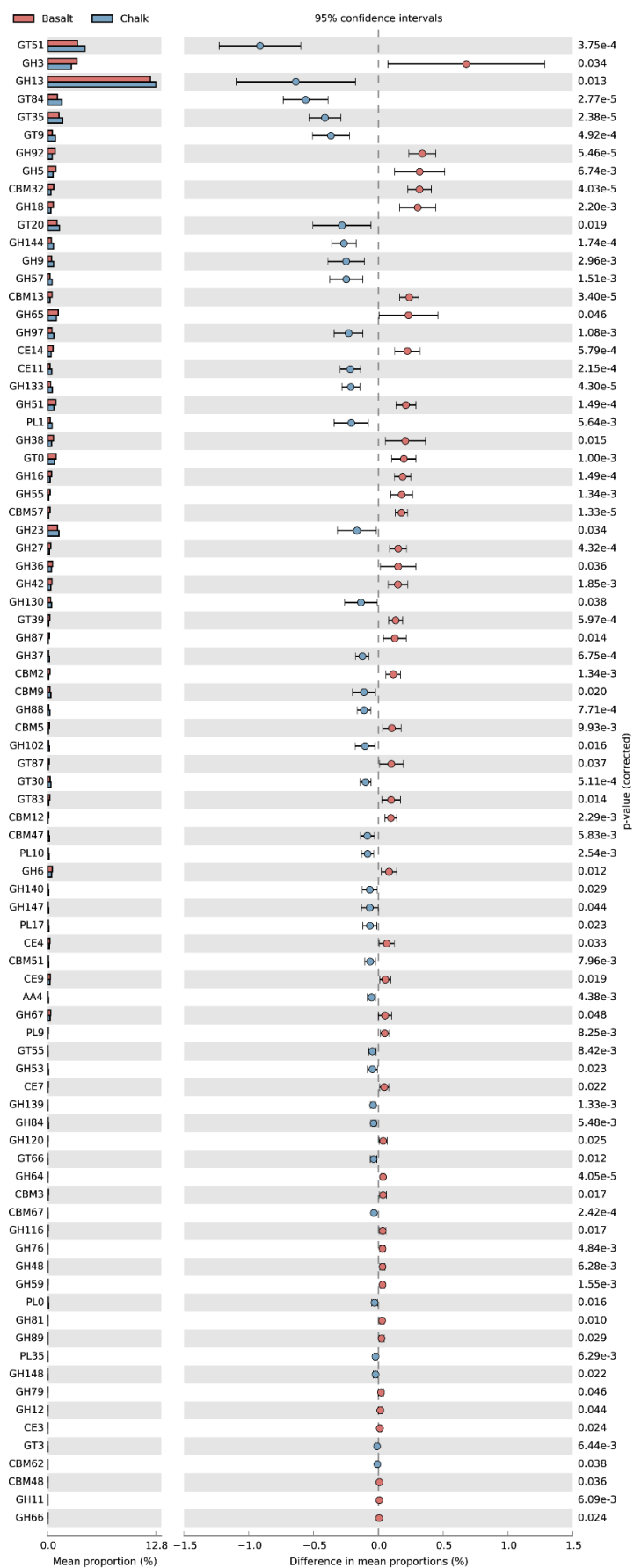

**Figure S2:** Level of significance for relative abundances of all CaZy in soil samples.

Supplement: Supplementary file 1 [file biology-11-01110-s001.zip › Supplementary Figure S3.pdf]

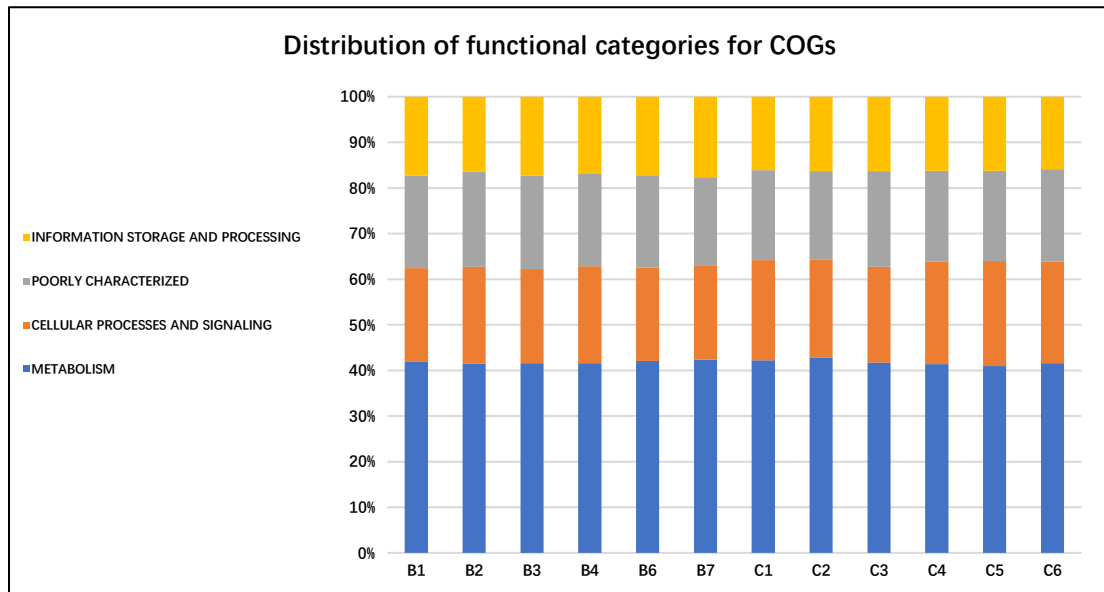

**Figure S3.** functional categories for COGs

Supplement: Supplementary file 1 [file biology-11-01110-s001.zip › Supplementary Figure S4.pdf]

# *Paenibacillus larvae*

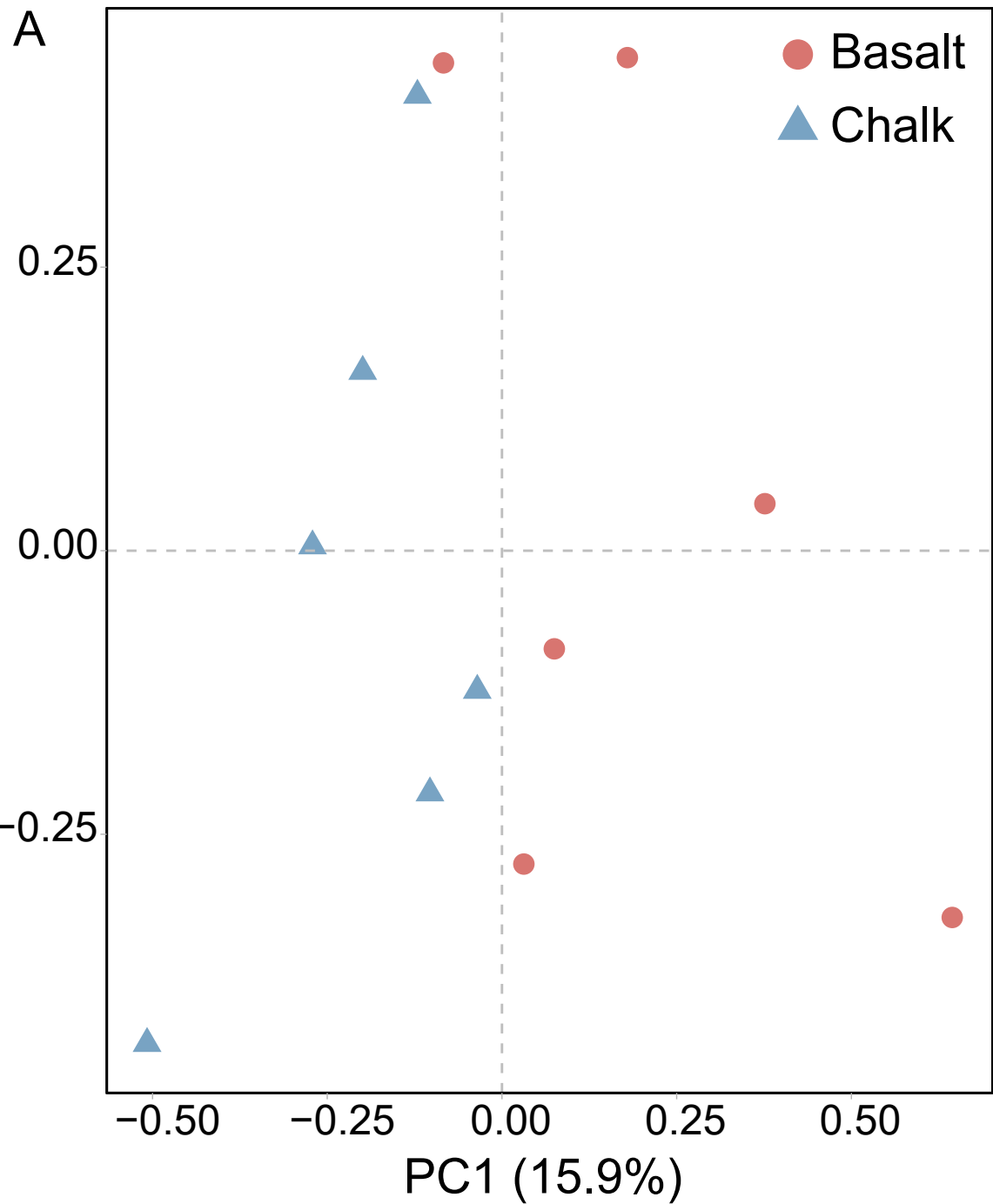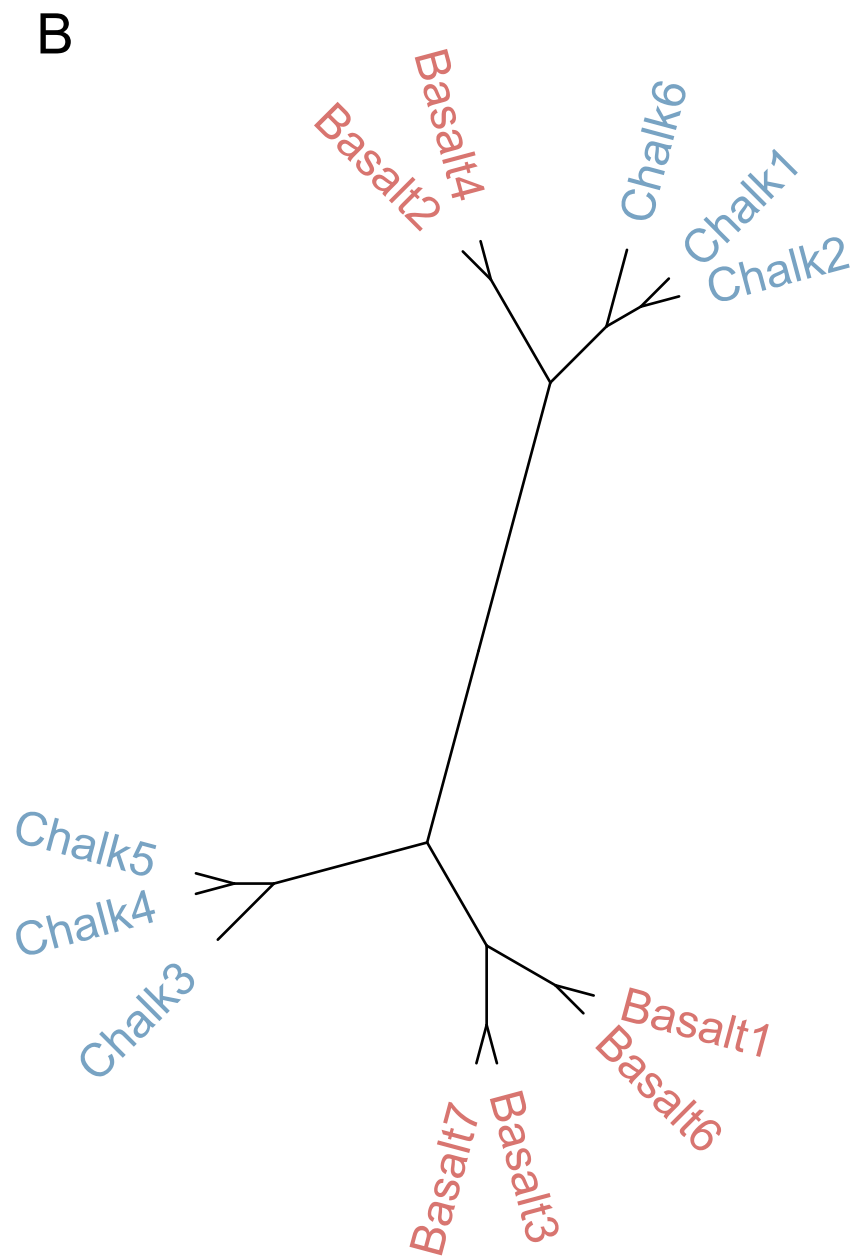

Supplement: Supplementary file 1 [file biology-11-01110-s001.zip › Supplementary Figure S8.pdf]

# *Conexibacter woesei*

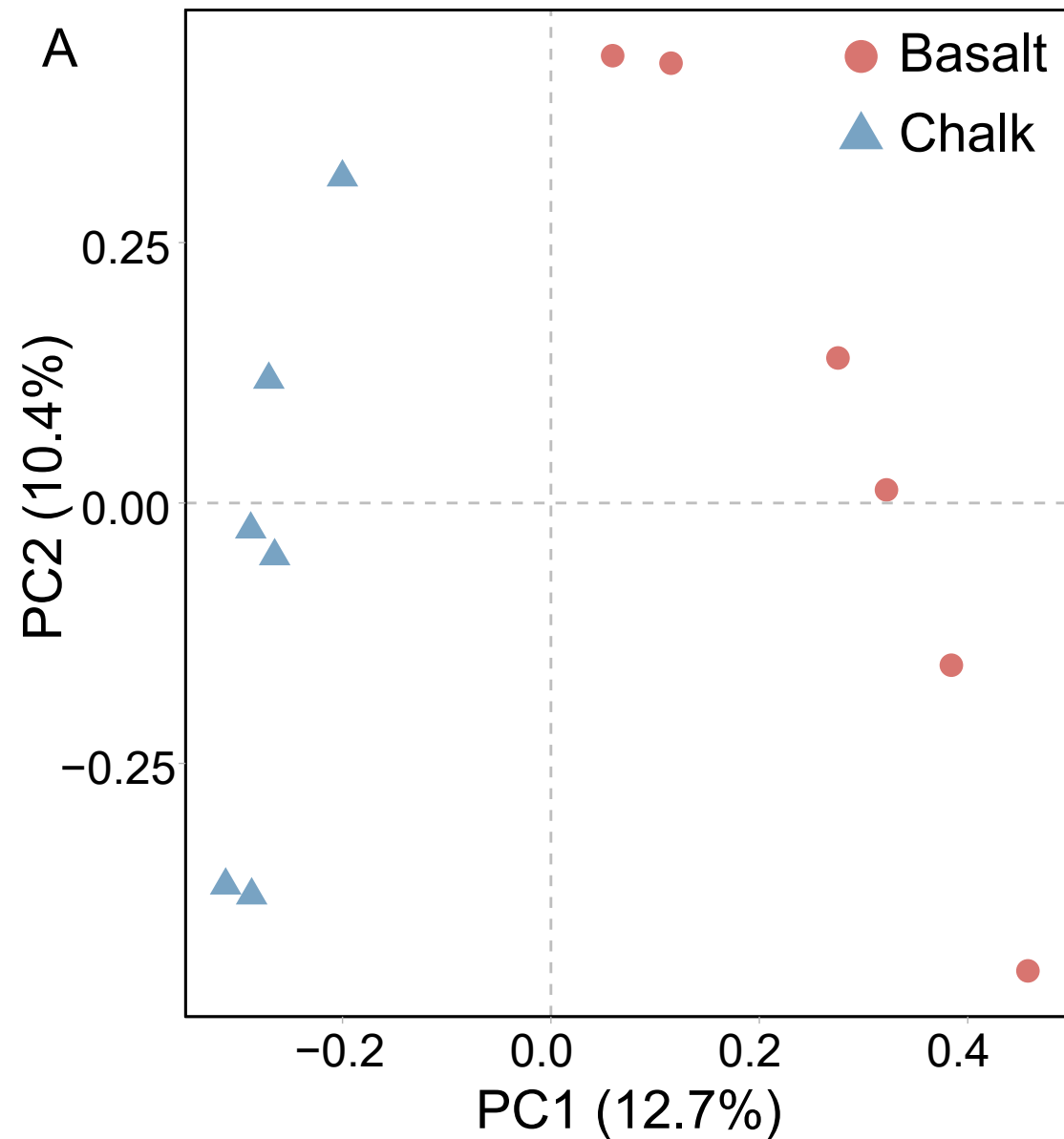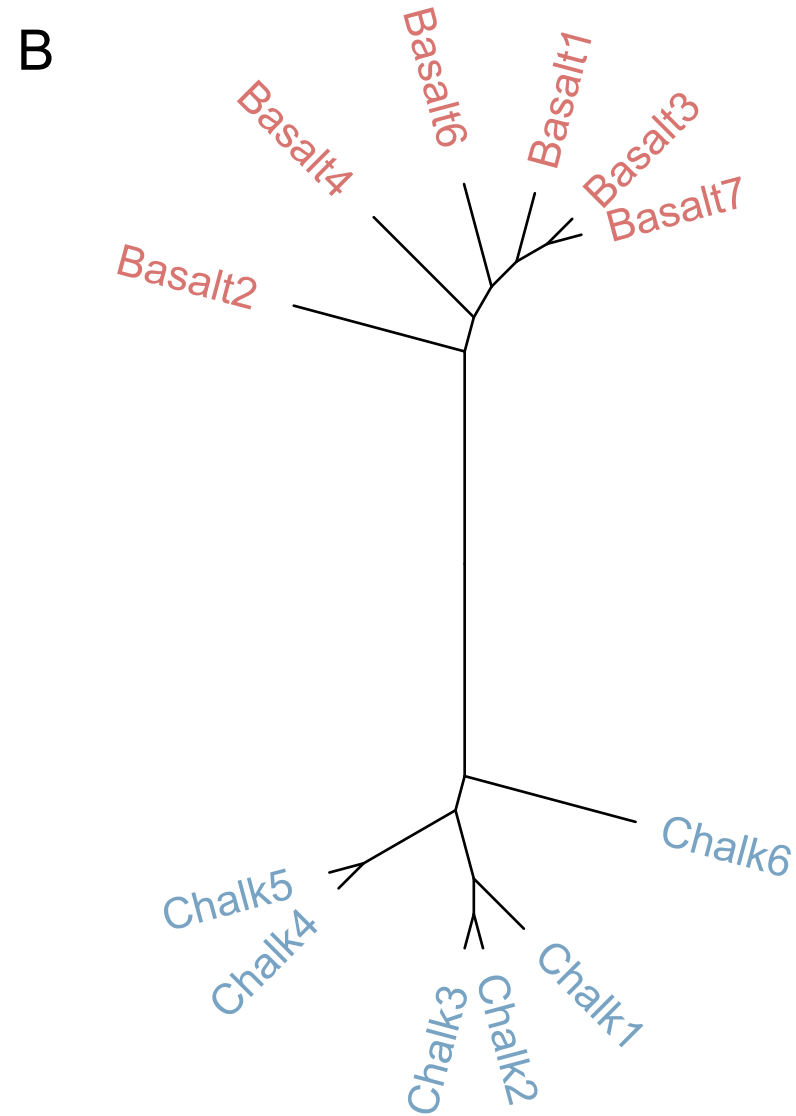

Supplement: Supplementary file 1 [file biology-11-01110-s001.zip › Supplementary Figure S9.pdf]
